# Supplementary material for: Permafrost landsystems define regional variability in climate change effects on northern environments
Source: Nat Commun. 2026 Apr 3;17:4799. doi: 10.1038/s41467-026-71216-2 (PMC13219417; doi:10.1038/s41467-026-71216-2)
Supplement: Supplementary file 1 — Supplementary Information [file 41467_2026_71216_MOESM1_ESM.pdf]

Permafrost landsystems define regional variability in climate change effects on northern environments

Steven V. Kokelj[1*], Stephen A. Wolfe[2], Niels Weiss[1], Duane Froese[3], Jennifer L. Baltzer[4], Trevor C. Lantz[5], H. Brendan O'Neill[2], Peter D. Morse[2], Anastasia Sniderhan[4], Niek J. Speetjens[5], Jurjen Van der Sluijs[6], Alejandro Alvarez[3], Suzanne E. Tank[7] & Stephan Gruber[8]

[1] Northwest Territories Geological Survey, Government of Northwest Territories, Yellowknife, NT, Canada
[2] Geological Survey of Canada, Natural Resources Canada, Ottawa, ON, Canada
[3] Department of Earth and Atmospheric Sciences, University of Alberta, Edmonton, AB, Canada
[4] Department of Biology, Wilfrid Laurier University, Waterloo, ON, Canada
[5] School of Environmental Studies, University of Victoria, Victoria, BC, Canada
[6] Northwest Territories Centre for Geomatics, Government of Northwest Territories, Yellowknife, NT, Canada
[7] Department of Biological Sciences, University of Alberta, Edmonton, AB, Canada
[8] Department of Geography and Environmental Studies, Carleton University, Ottawa, ON, Canada
* Corresponding Author. Email: Steve_Kokelj@gov.nt.ca

Supplementary information

Supplementary Figure 1. Permafrost landforms that reveal ground ice conditions have evolved with ecosystems to indicate landscape vulnerability to thaw

Supplementary Table 1. Thermokarst and periglacial landforms inventoried for the study region

Supplementary Table 2. The Northwest Territories Land Classification Framework and labeling used in this study

Supplementary Table 3. Redundancy analysis variable descriptions

Supplementary Appendix 1. Oblique and satellite imagery of characteristic landforms and landscapes for Level IV Ecoregions

References

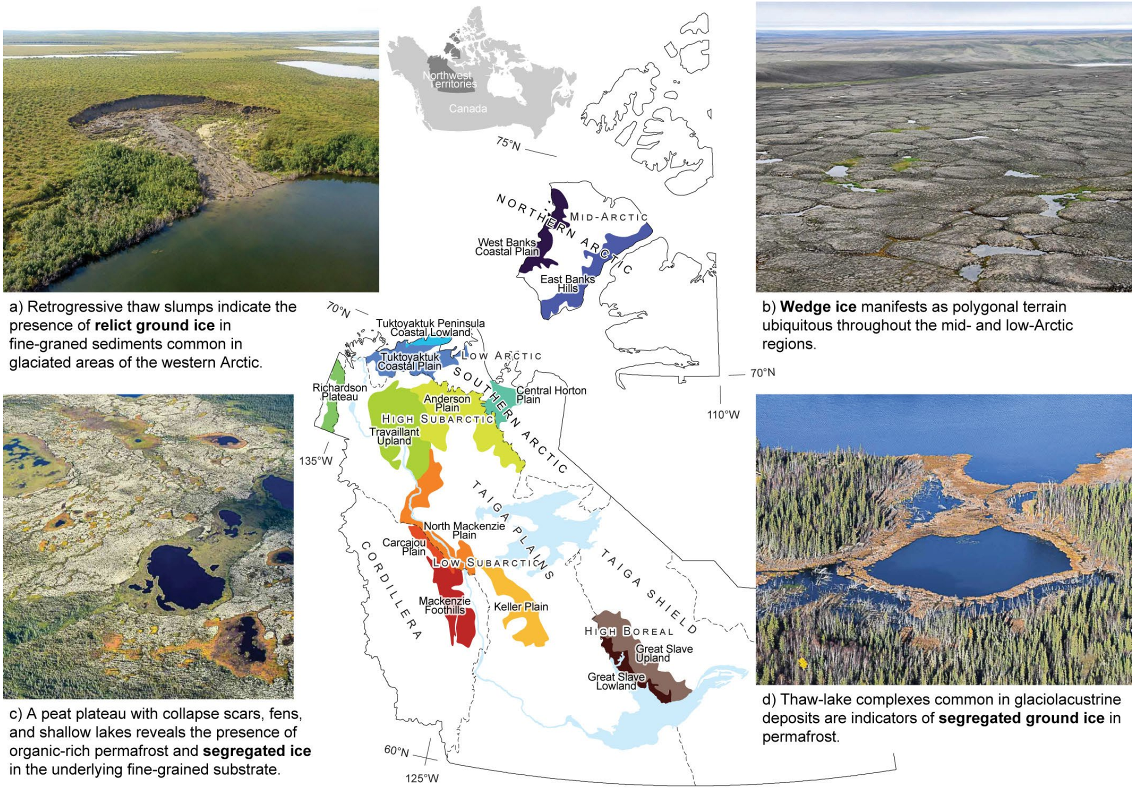

Supplementary Figure 1. Permafrost landforms that reveal ground ice conditions have evolved with ecosystems to indicate landscape vulnerability to thaw. a) is from the Tuktoyaktuk Coastal Plain, b) is from the East Banks Hills, c) is from the North Mackenzie Plain, and d) is from the Great Slave Lowland. The Taiga Plains, Cordillera, Shield, Tundra Cordillera, and the Southern and Northern Arctic are the major ecozones within the study region. From these ecozones, we examine landform assemblages of 14 Level IV ecoregions, mapped by the NWT Thermokarst Mapping Collective[1]. Map adapted from Level IV Ecoregions[2–6]. Ecoregion outlines and other spatial layers derived from https://www.apps.geomatics.gov.nt.ca/arcgis/rest/services

Supplementary Figure 1 – Supporting information on permafrost ground-ice types

This section briefly describes common permafrost ground-ice types that yield ice volumes greater than the pore space of the hosting substrate, referred to as excess ice. The development of ground ice can produce distinctive periglacial landforms, such as polygonal terrain, in areas underlain by ice wedges. Thawing permafrost containing excess ground ice will produce surface subsidence, terrain modification, and the development of characteristic "thermokarst" landforms. For the purpose of this paper, we collectively refer to these as "Permafrost landforms". For additional details on ground-ice terminology, refer to Lewkowicz et al.[7] and references within.

Segregated ice consists of lenses or layers of ice that form due to temperature-induced migration of pore water in freezing soil. Segregated ice forms in saturated fine-grained soils at depth when permafrost establishes and in the near-surface when sedimentation or vegetation succession causes the upward aggradation of the permafrost table.

Landform indicators of segregated ice include lithalsas and permafrost peat plateaus. Indicators of thawing segregated ice can include shallow slides and slumps, thaw lakes, expansion and collapsing shorelines, and slumping. Indicators of thawing segregated ice beneath permafrost peatlands are collapse scars or larger collapse basins occupied by bogs and fens.

Relict ice refers to ground ice that has formed in a past environment that could not develop in its stratigraphic position under the current environmental conditions. In the Northwest Territories, it is most commonly in the form of buried glacial ice associated with moraine belts, but it also occurs under outwash deposits.

Permafrost landform indicators of thawing relict ice include retrogressive thaw slumps, mega slumps, moraine lakes and slumps, and lake expansion or drainage. Intrusive ice is indicated by involuted terrain, and its thawing manifests as expanding thaw ponds and retrogressive thaw slumps.

Wedge ice develops due to thermal contraction cracking of the ground and infilling with meltwater to form a vein of ice. Repeated cracking over millennia can cause a large ice wedge to develop. Thermal contraction cracking of permafrost requires cold winter air and near-surface ground temperatures.

The surface expression of an ice-wedge network is polygonal terrain common across tundra environments underlain by unconsolidated sediments. Patterned forests indicate polygonal terrain in some alluvial deposits. Polygonal patterned ponds within troughs or polygon centres also indicate the presence of wedge ice, and rapid increases in pond size indicate its thawing.

Supplementary Table 1. Thermokarst and periglacial landforms inventoried for the study region. For the purposes of this paper, together we refer to these as Permafrost landforms that combine to form *permafrost landform assemblages*.

**Landscape feature and *Code***	**Photograph**	**Description and dominant ground ice and soil associations**
Retrogressive thaw slump<br><br>*RTS*	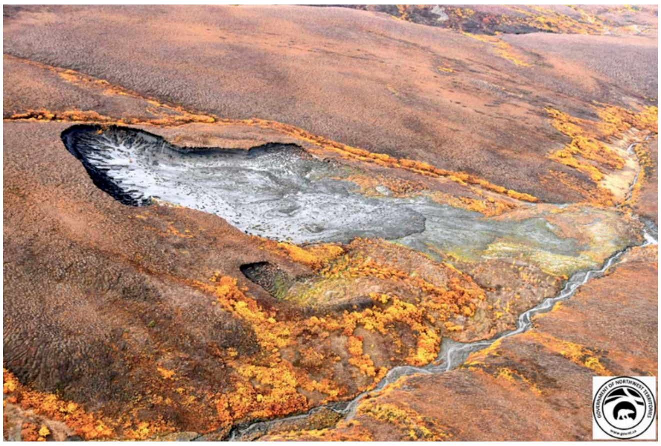<br>20210914_181351_NIKON_BDL_0733.jpg	**Description:** Permafrost landslide comprised of a thawing, near vertical ice-rich headwall, a scar area comprised of thawed slurry, and a debris lobe of materials that have gradually flowed downslope.<br>**Ground ice:** Relict, Segregated<br>**Substrate:** Mineral
Mega slump<br><br>*Megaslump*	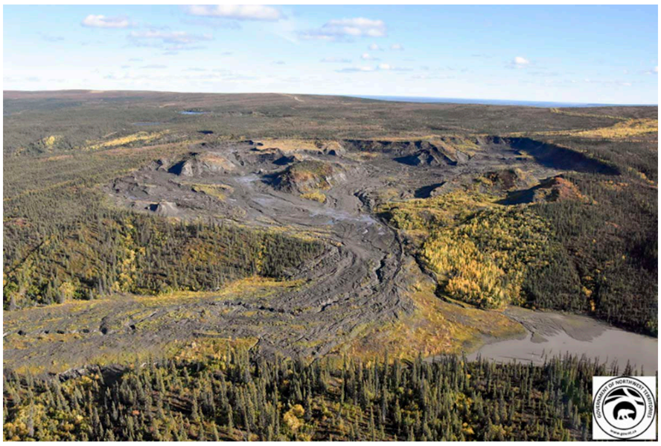<br>20220911_174822_NIKON_D5600_BDL_0283.jpg	**Description:** Primary processes are similar to a retrogressive thaw slump, but processes of downslope sediment evacuation become increasingly dominant, driving further downwasting, in turn perpetuating upslope growth, leading to nodes of major slope and downstream modification.<br>**Ground ice:** Relict, Segregated<br>**Substrate:** Mineral
Shallow landslide<br><br>*Landslide_ Shallow*	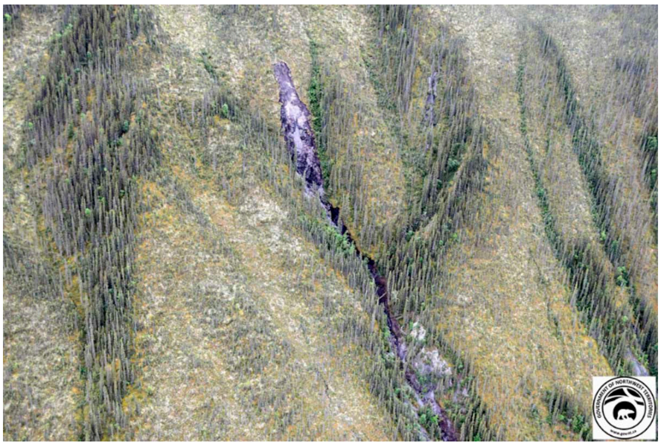<br>20220712_110724_NIKON_D5600_BDL_0670.jpg	**Description:** Elongated shallow slides on sloping terrain. Transport is constrained to materials within the active layer. The absence of a large debris tongue indicates a smaller volume of material transported.<br>**Ground ice:** Segregated<br>**Substrate:** Mineral

Deep translational landslide<br><br>*Landslide_Deep*	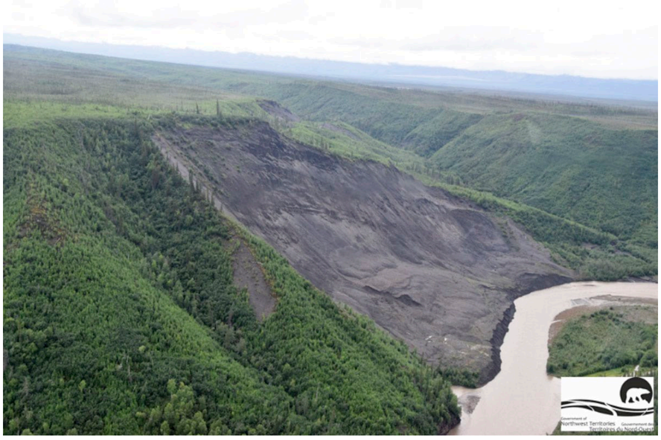<br>20200805_125739_NIKON_NWL_0016.jpg	**Description:** Large failures with cuspate-shaped scars and downslope debris deposits indicate a high volume of translocated materials. Failure plain may be related to the substrate or at the base of thin permafrost.<br>**Ground ice:** Segregated, Pore<br>**Substrate:** Mineral, Bedrock
Active colluvial fan<br><br>*Debris_Flow_Fan*	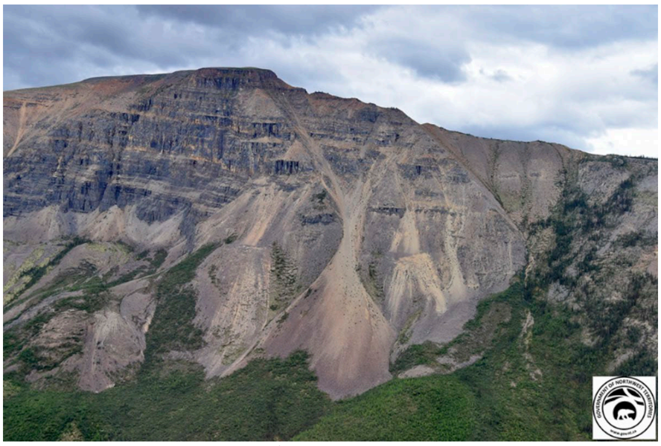<br>20220712_122818_NIKON_D5600_BDL_0838.jpg	**Description:** Evidence of recent runout of flow over colluvial fan or colluvial slope, typically in mountainous terrain.<br>**Ground ice:** Pore, Segregated<br>**Substrate:** Regolith
Gullied terrain<br><br>*Gullied_Terrain*	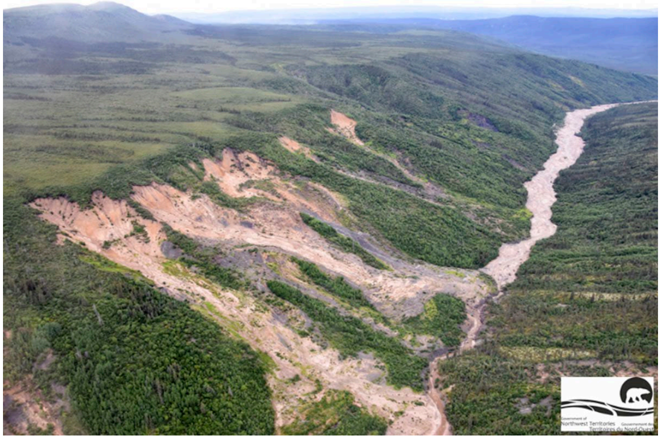<br>20200807_100239_NIKON_NWL_0085.jpg	**Description:** The distinct slope morphological pattern of some incised valleys typically indicates a legacy of shallow sliding. Here gullied terrain and slides occur along the same valley slope.<br>**Ground ice:** Segregated, Pore<br>**Substrate:** Mineral/Colluvium

Rock glaciers<br><br>*Rock_Glaciers*	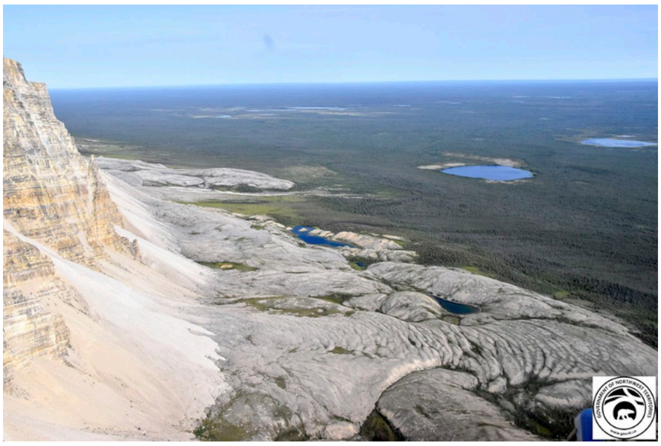<br>20210717_104042_NIKON_NSL_0074.jpg	**Description:** Ice and frozen sediments covered by seasonally frozen rock debris, showing evidence of creep deformation of the talus slope.<br>**Ground ice:** Pore, Segregated<br>**Substrate:** Regolith
Slump-affected lake<br><br>*Slump_Lakes*	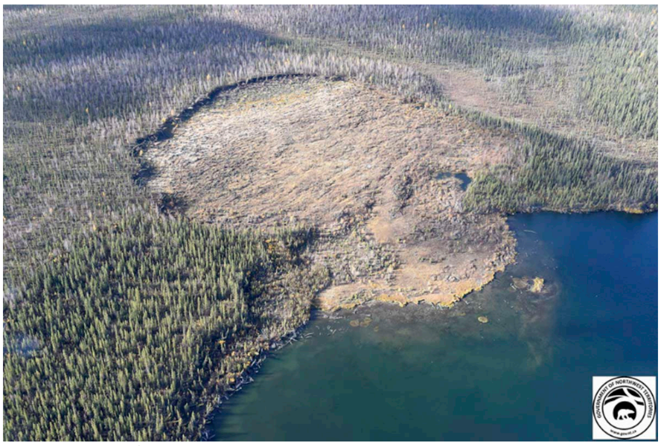<br>20210914_125121_NIKON_BDL_0191.jpg	**Description:** Lake or pond with shoreline affected by RTS.<br>**Ground ice:** Relict, Segregated<br>**Substrate:** Mineral
Lake drainage<br><br>*Lake_Drainage*	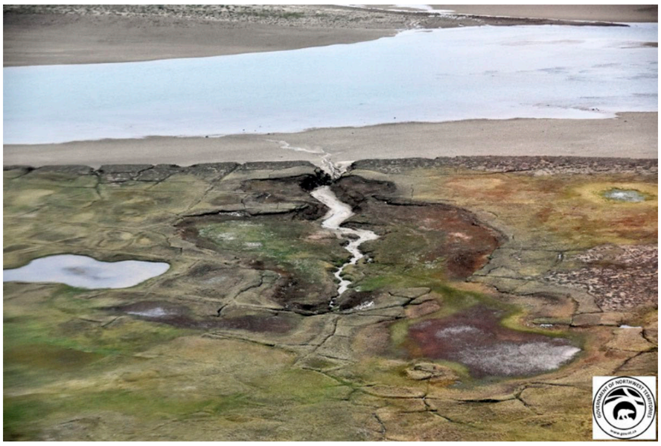<br>20210803_161614_NIKON_NSL_0637.jpg	**Description:** The gullied outflow of this drained lake basin shows evidence of rapid drainage.<br>**Ground ice:** Segregated, Wedge<br>**Substrate:** Mineral, Organic

Lowered water level<br><br>*Lowered_Water_ Level*	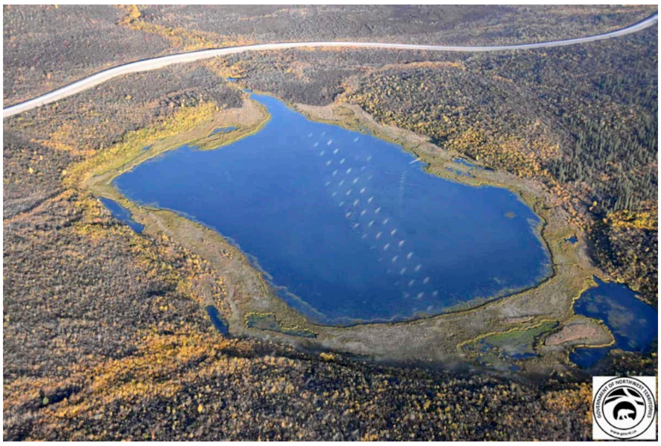<br>20210909_100110_NIKON_BDL_0010.jpg	**Description:** Any lake showing evidence of recent lowering where the cause can not be determined confidently. The lowered level may be due to drainage or drying, with the latter typically showing a regional pattern.<br>**Ground ice:** Variable<br>**Substrate:** Mineral, Organic
Ramparted lake<br><br>*Ramparted_Lake*	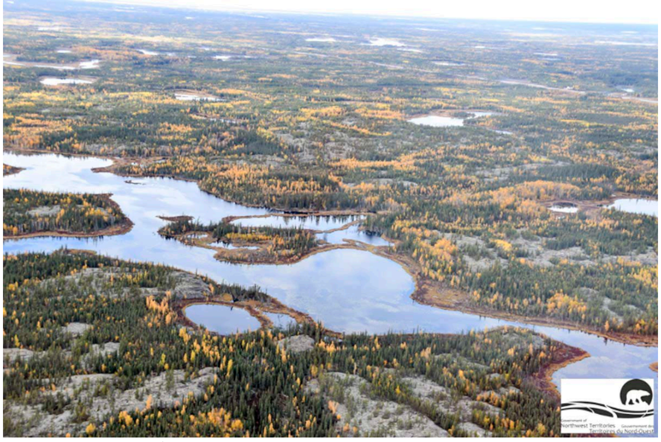<br>20200921_172954_NIKON_NSL_0186.jpg	**Description:** These irregularly shaped lakes are associated with numerous ponds and adjacent undulating terrestrial ridges. These lake-terrestrial associations occur as ramparted lake-lithalsa complexes in glaciolacustrine deposits and in shallow basins in organic terrain.<br>**Ground ice:** Segregated<br>**Substrate:** Mineral
Polygonal Patterned ponding<br><br>*Patterned_Pondin g_Polygonal*	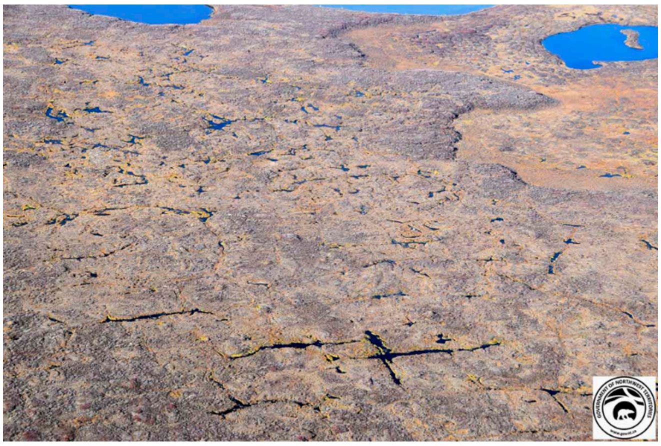<br>20210909_173405_NIKON_BDL_0731.jpg	**Description:** Includes trough ponds in low-centred polygons and larger coalesced ponds. Upland trough ponds are increasingly visible in low and mid-Arctic tundra due to top-down ice-wedge thaw.<br>**Ground ice:** Wedge<br>**Substrate:** Mineral, Organic

Thermokarst ponds<br><br>*Patterned_Ponding_other*	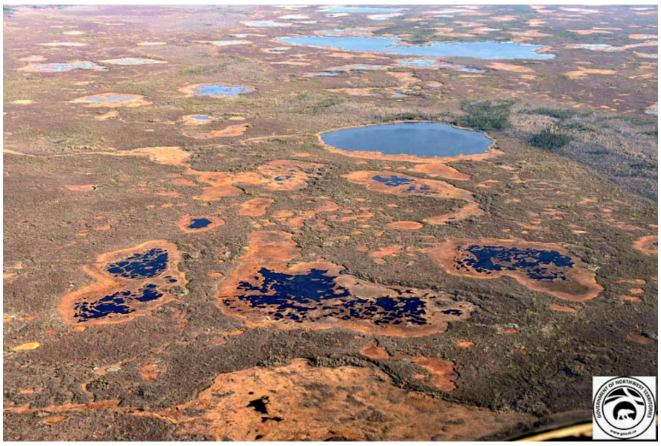<br>20220928_154943_NIKON_D5600_BDL_0378.jpg	**Description:** Small ponds typically related to thermokarst lakes or wetlands where other indicators of ice-rich terrain are observed (i.e.: Ramparted lake-lithalsa complexes, collapse scars, and basins in peatlands).<br>**Ground ice:** Segregated<br>**Substrate:** Mineral, Organic
Geometric lakes<br><br>*Geometrically_Aligned_Lakes*	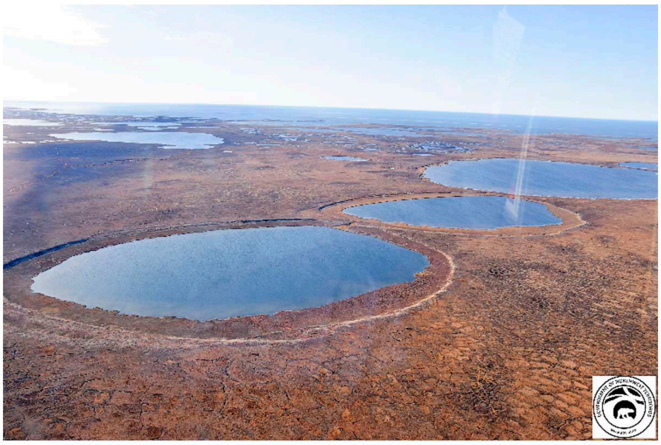<br>20210909_174308_NIKON_BDL_0777.jpg	**Description:** Includes oriented lakes and smaller, shallow water bodies associated with the coalescence of low-centred polygonal ponds in mid-Arctic environments.<br>**Ground ice:** Variable<br>**Substrate:** Mineral
Littoral terraces<br><br>*Littoral_Terrace_Count*	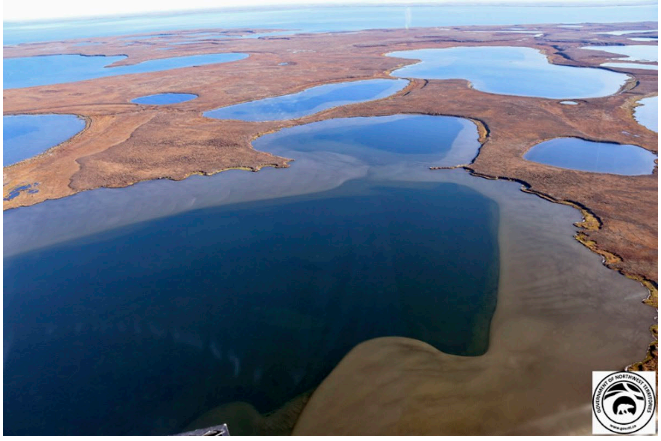<br>20210909_180436_NIKON_BDL_0912.jpg	**Description:** Lakes with prominent littoral terraces around shorelines are often associated with oriented lakes. Permafrost is typically present beneath the shallow terraces where lake ice is bottom fast.<br>**Ground ice:** Pore, Segregated<br>**Substrate:** Mineral (sandy)

Expansion or development of lakes and ponds<br><br>*Lake_Pond_Expansion*	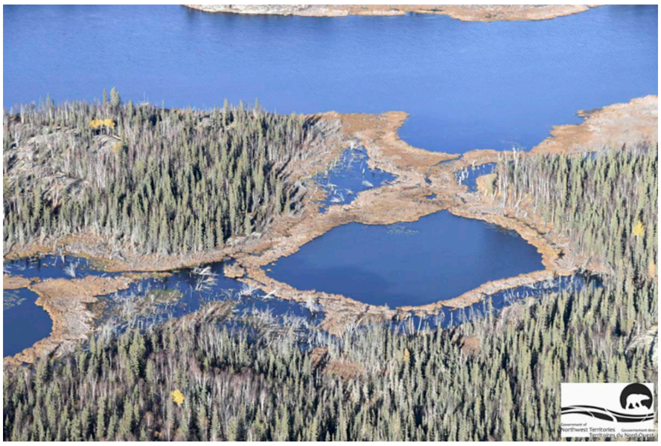<br>20200928_162919_NIKON_NSL_0230.jpg	**Description:** Lakes or ponds developed or enlarged between SPOT 4/5 (2004/05) and Sentinel-2 (2017/18) imagery. Typically, an attribute associated with patterned ponds undifferentiated, collapse scars/basins, or Ramparted Lake-lithalsa complexes.<br>**Ground ice:** Segregated, Relict<br>**Substrate:** Mineral, Organic
Expansion or development of polygonal patterned ponds<br><br>*Patterned_Ponding_Expansion*	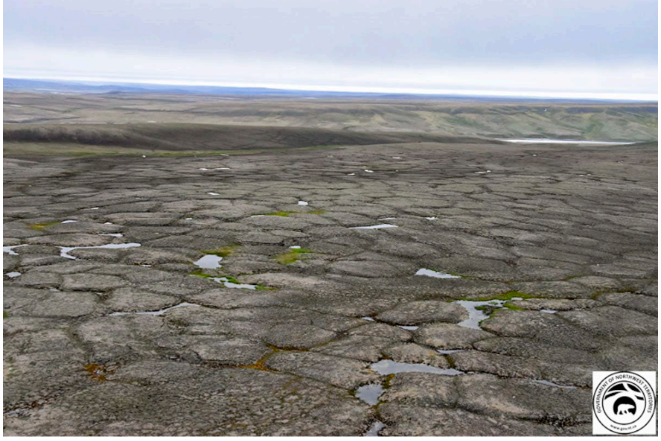<br>20210804_174947_NIKON_NSL_0828.jpg	**Description:** Patterned ponds associated with polygonal terrain developed or enlarged between SPOT 4/5 (2004/05) and Sentinel-2 (2017/18) imagery.<br>**Ground ice:** Wedge<br>**Substrate:** Mineral, Organic
Polygonal terrain<br><br>*Total_Polygonal_Terrain*	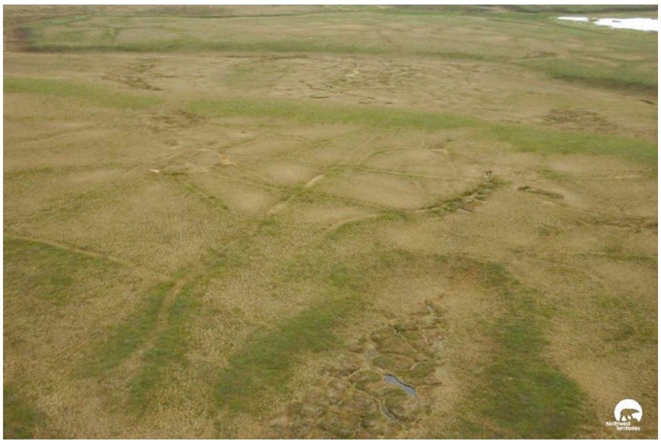<br>NWT2009-07-16DSC_0450.JPG	**Description:** A combination of all indicators of polygonal terrain on a grid cell.<br>**Ground ice:** Wedge<br>**Substrate:** Mineral, Organic

Upland polygonal terrain<br><br>*Polygonal_Terrain _Upland*	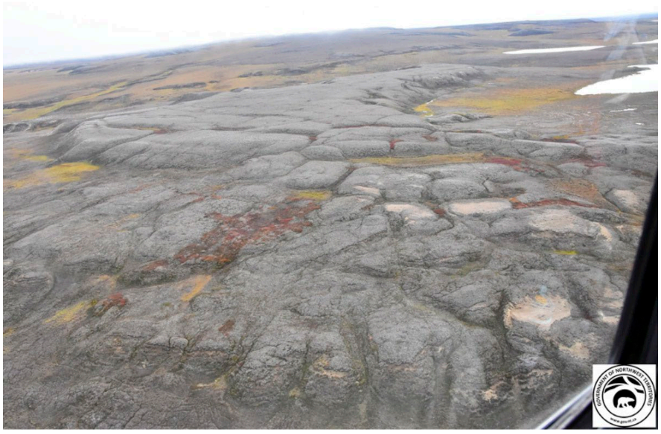<br>20210828_153111_NIKON_NSL_0600.jpg	**Description:** A combination of all indicators of polygonal terrain on hilltops and valley slopes.<br>**Ground ice:** Wedge<br>**Substrate:** Mineral
Lowland polygonal terrain<br><br>*Polygonal_Terrain _Lowland*<br>and<br>*Polygonal_Terrain _Floodplain*	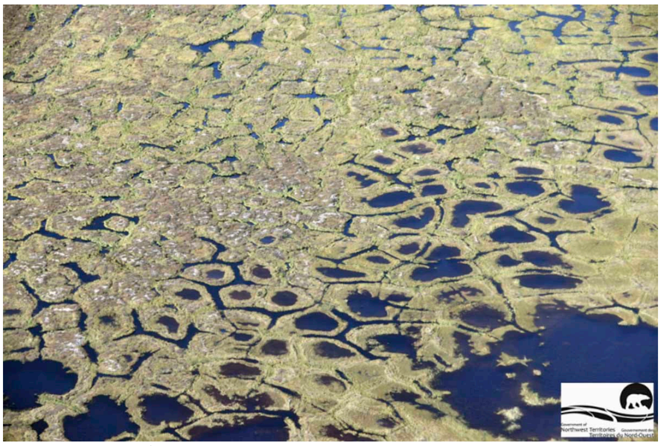<br>20200822_170520_NIKON_INU_0378.jpg	**Description:** A combination of all indicators of polygonal terrain in lowlands and floodplains.<br>**Ground ice:** Wedge<br>**Substrate:** Organic, Mineral
Patterned forests in lowland areas<br><br>*Patterned_Forest_ Lowland*	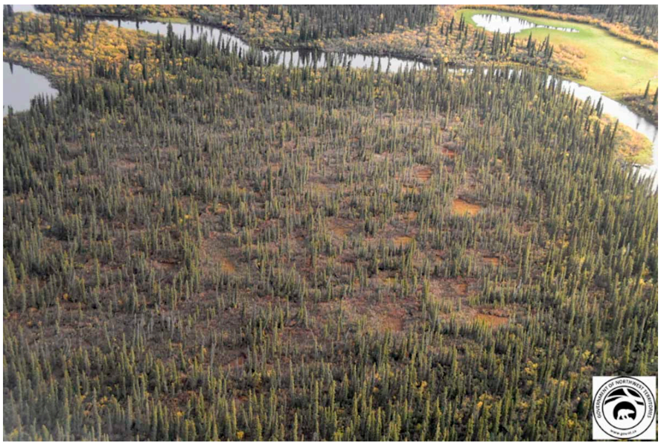<br>20220911_164747_NIKON_D5600_BDL_0165.jpg	**Description:** Typically, spruce trees in alluvial deposits with ice wedges grow preferentially close to the wedges, leading to a patterned forest cover.<br>**Ground ice:** Wedge (continuous), Segregated (discontinuous)<br>**Substrate:** Mineral, Organic in discontinuous

Collapse scars in organic terrain<br><br>*Organic_Collapse_Scars*	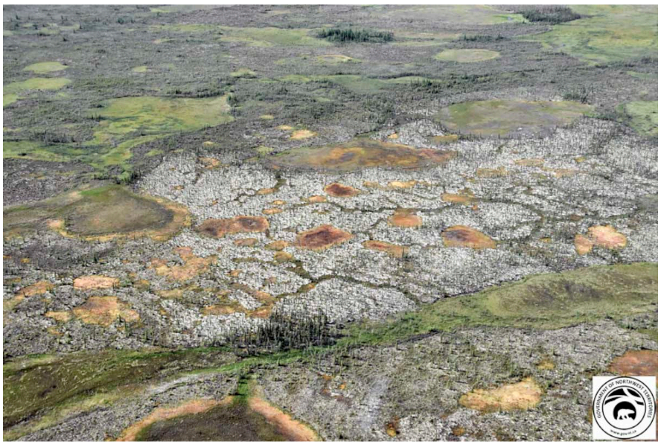<br>20210717_123002_NIKON_NSL_0109.jpg	**Description:** In permafrost peatlands, bogs, ponds, or larger dry basins typically lack near-surface permafrost. These landforms are enlarging across discontinuous permafrost with climate-driven warming. These landforms are inventoried under the code "Organic_Collapse_Scars" but generally comprise "permafrost free bogs, fens, ponds and shallow lakes" within a permafrost peatland.<br>**Ground ice:** Segregated (in adjacent peatland)<br>**Substrate:** Organic
Dendritic drainage networks in organic terrain<br><br>*Organic_Drainage_Dendritic*	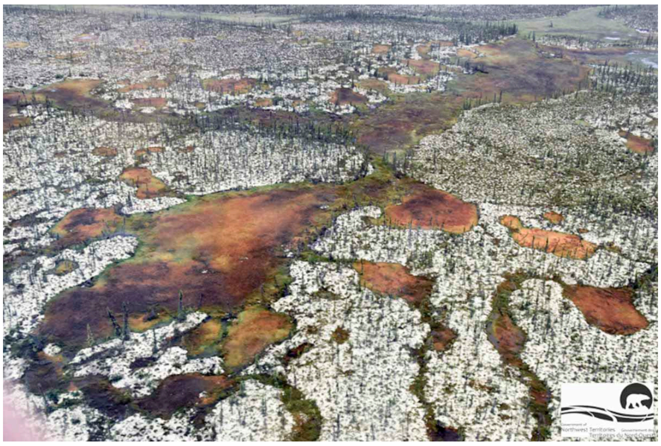<br>20200804_161521_NIKON_NWL_0186.jpg	**Description:** Refers to the dendritic drainage pattern within permafrost peatlands, typically associated with fen networks that drain gently sloping organic terrain in till plains.<br>**Ground ice:** Segregated (in adjacent peatland)<br>**Substrate:** Organic
Rectangular drainage networks in organic terrain<br><br>*Organic_Drainage_Rectangular*	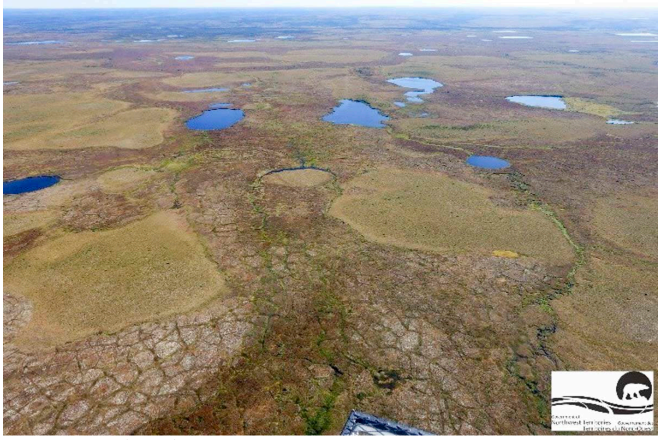<br>20200822_151715_SONY_DSC03992.jpg	**Description:** Refers to the angular drainage network within permafrost peatlands, typically associated with the degradation of an underlying ice-wedge network.<br>**Ground ice:** Wedge<br>**Substrate:** Organic

Multibasinal drainage networks in organic terrain<br><br>*Organic_Drainage_MBC*	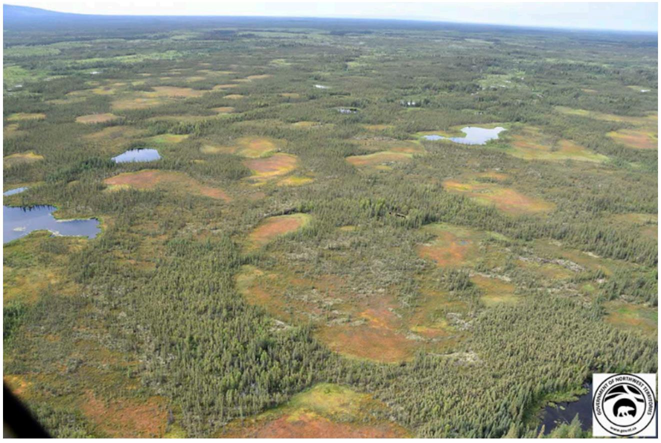<br>20220712_170741_NIKON_D5600_BDL_0205.jpg	**Description:** Refers to patterned or thermokarst ponds or basins within permafrost peatlands. In discontinuous permafrost, this drainage network is typically associated with small bogs & ponds or larger basins & shallow lakes. In the zone of continuous permafrost, this drainage network is generally associated with polygonal patterned ponding in either troughs or polygon centres, where it may also be linked with rectangular drainage patterns.<br>**Ground ice:** Segregated<br>**Substrate:** Organic
Patterned fens in organic terrain<br><br>*String_Bog*	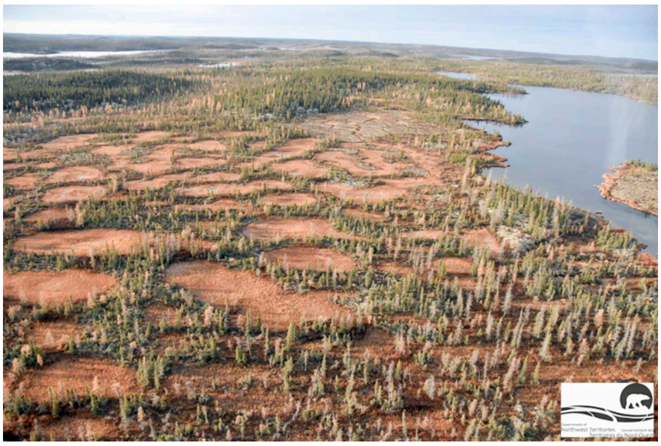<br>20201002_092614_NIKON_NSL_0112.jpg	**Description:** Patterned fens are common within drainage networks for permafrost peatlands in the discontinuous and southern discontinuous permafrost zones. However, they are also observed in organic terrain across the treeline transition zone.<br>**Ground ice:** Segregated<br>**Substrate:** Organic
Pingos & conical mounds<br><br>*Pingo_Count*	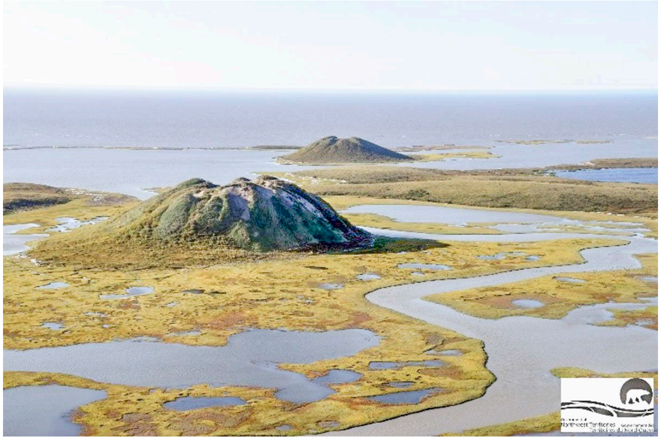<br>20200821_191425_NIKON_INU_0298.jpg	**Description:** Ice-cored conical hills are most common in drained lake basins. Areas of uplifted terrain due to segregated ice accumulation do not fit the strict definition of a pingo. Still, they may be common in parts of the western Arctic and inventoried under the "Pingo" class.<br>**Ground ice:** Intrusive, Segregated<br>**Substrate:** Mineral (sandy)

<table>
<tr>
<td>Involuted terrain

Involuted_Terrain</td>
<td>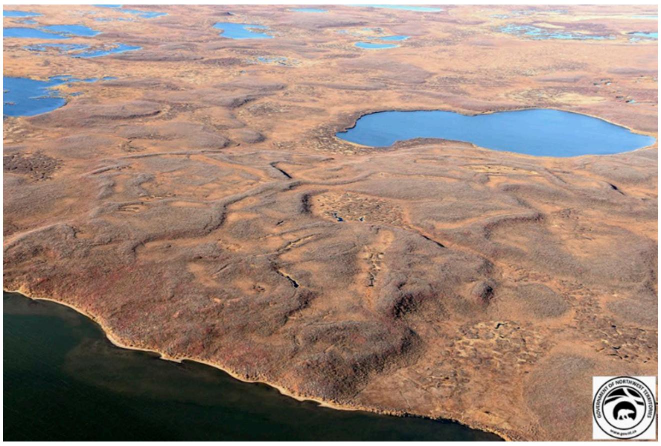
20210909_172741_NIKON_BDL_0693.jpg</td>
<td>Description: This ice-cored terrain is comprised of intrusive segregated ice. Hills typically have gentle ridges, which give the appearance of a wrinkled prune.
Ground ice: Intrusive, Segregated
Substrate: Mineral, (Sandy)</td>
</tr>
</table>

Supplementary Table 2. The Northwest Territories Land Classification Framework and labeling used in this study, as well as the numbers of grid cells in each Level IV ecoregion and those used in the Redundancy Analysis[2–6]. MA is Mid-Arctic, LAn is Low Arctic north, HS is High Subarctic, LS is Low Subarctic, and HB is High Boreal.

BIOME (Level I Ecoregion)	ECOZONE (Level II Ecoregion)	ECOCLIMATE REGION (Level III Ecoregion)	FIGURE 4. (Level II & III Ecoregion)	LANDSYSTEM (Level IV Ecoregion)	Gridcells in RDA (n=1959)	Total gridcells (n=3292)
Tundra	Northern Arctic	Mid-Arctic	Northern Arctic MA	West Banks Coastal Plain MA	98	197
				East Banks Hills MA	250	286
	Southern Arctic: Tundra Plains	Low Arctic north	Southern Arctic LAn	Tuktoyaktuk Peninsula Coastal Lowland LAn	36	62
				Tuktoyaktuk Coastal Plain LAn	150	288
				Central Horton Plain LAn	101	122
	Tundra Cordillera	High Subarctic	Tundra Cordillera HS	Richardson Plateau HSas	27	87
Taiga	Taiga Plains		Taiga Plains HS	Travaillant Upland HS	375	456
				Anderson Plain HS	121	522
	Taiga Cordillera	Low Subarctic	Taiga Cordillera LS	Mackenzie Foothills LSbs	69	249
				Carcajou Plain LSb	48	49
	Taiga Plains		Taiga Plains LS	North Mackenzie Plain LS	263	280
				Keller Plain LS	251	256
	Taiga Shield	High Boreal	Taiga Shield HB	Great Slave Lowland HB	100	166
				Great Slave Upland HB	70	272

Supplementary Table 3. Redundancy analysis variable descriptions. The eight independent environmental variables retained in the redundancy analysis to examine their associations with landform assemblages for a total of 1959 grid cells from across 14 level IV ecoregions. Additional derived or modeled variables used in testing are in italics at the bottom of the table. Testing revealed the high collinearity between the primary and derived variables, and their addition improved the total explained variation of the RDA by less than 2%.

**Primary variables**	**Description**	**Unit**	**Source**
MAAT	Thirty-year mean of 2 m Mean Annual Air Temperature (1991-2020)	°C	Muñoz-Sabater, 2019[8]
Rainfall	Thirty-year mean of total annual liquid precipitation (1991-2020)	m	Muñoz-Sabater, 2019[8]
SWE	Thirty-year mean of total annual max of monthly Snow Water Equivalent (1991-2020)	m	Muñoz-Sabater, 2019[8]
Burn area	Total area per grid cell burnt by wildfire between 1996 and 2016	m^2	Government of Northwest Territories (GNWT). Fire History[9]
Geomorphon	Geomorphon (terrain position classifications) variety; how many different geomorphon features are found in each grid cell	-	Amatulli et al., 2020[10]
HAND	Mean Height Above Nearest Drainage (relative elevation between the grid cell and its nearest water stream)	m	Donchyts et al., 2016[11]
Flow accumulation	Flow accumulation	km^2	Amatulli et al., 2022[12]
Percent water	Part of each grid cell covered by water	%	Topographic Data of Canada - CanVec Series. Lakes, Rivers and Glaciers in Canada - Hydrographic Features - 1:250k[13]
**Derived or modeled variables use in testing but NOT included in final analysis**			
Solar_rad	*Area Solar Radiation*	*WH m^{-2}*	*2016 total annual incoming solar radiation, calculated using ArcMap Area Solar Radiation tool in Spatial Analyst toolbox. DEM [14] resampled to 100 m resolution, for entire year in 2016, ran for each degree latitude*
Mean Water Body	*Mean water body size within each grid cell*	*km^2*	Topographic Data of Canada - CanVec Series. Lakes, Rivers and Glaciers in Canada - Hydrographic Features - 1:250k[13]
Modeled Relict ice	*Mean quantitative value within grid cell*	*0-4*	*O'Neill et al., 2019*[15]
Modeled Segregated ice	*Mean quantitative value within grid cell*	*0-4*	*O'Neill et al., 2019*[15]
Modeled Wedge ice	*Mean quantitative value within grid cell*	*0-4*	*O'Neill et al., 2019*[15]
Ground ice sum	*Combined quantitative value within grid cell*	*0-12*	*O'Neill et al., 2019*[15]
Ground ice var	*Number of ground ice types within each grid cell*	*0-3*	*O'Neill et al., 2019*[15]

Landcover_dom	*Dominant landcover type*	-	
Landcover_var	*Landcover variety (how many different surficial geology types are found in each grid cell)*	-	
Geomorph_dom	*Dominant geomorphon*	-	*Amatulli et al., 2020*[10]
PF_prob	*Permafrost probability, averaged by grid cell*	%	*Obu et al., 2018*[16]
Surf_geol_var	*Surficial geology variety (how many different surficial geology types are found in each grid cell)*	-	*O'Neill et al., 2019*[15]
Surf_geol_dom	*Dominant surficial geology*	-	*O'Neill et al., 2019*[15]

Supplementary Appendix 1. Oblique and satellite imagery of characteristic landforms and landscapes for Level IV Ecoregions

Sentinel-2 and oblique images of landforms and landscapes that characterize the Level IV ecoregions shown in Figure 5 (b-e). Level IV ecoregions are presented in alphabetical order.

All Sentinel imagery from the European Space Agency, Copernicus Sentinel data.

Oblique photographic images are from the Northwest Territories Thermokarst Mapping Collective (Government of Northwest Territories, Northwest Territories Geological Survey; https://www.apps.geomatics.gov.nt.ca/arcgis/rest/services/GNWT_Operational/NTGS_ThermokarstCollective_Operational/MapServer) and the Ecological Land Classification Photo Inventory (Government of Northwest Territories, ECC, Fire Management Division; https://www.appstest.geomatics.gov.nt.ca/arcgis/rest/services/GNWT/BiologicEcologic_LCC/MapServer/3)

Inset maps show the Northwest Territories and Level IV ecoregions[2–6]. Outlines and other spatial layers adapted from https://www.apps.geomatics.gov.nt.ca/arcgis/rest/services

Compilation by V. Pauze

Anderson Plain (AP)

Taiga Plains - High Subarctic

29,808 km²

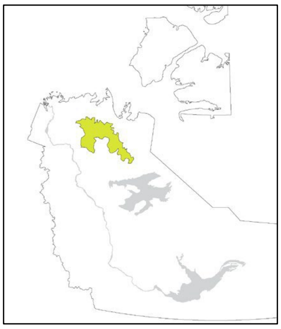

Sentinel-2 imagery of terrain aligned (glacially-fluted), geometric lakes in Anderson Plain.

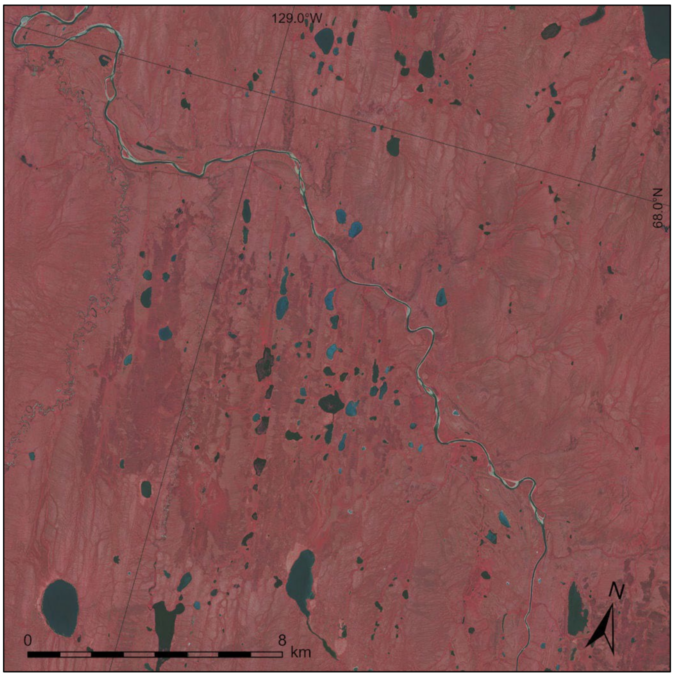

Sentinel-2 of lowland polygonal terrain (black arrows) in Anderson Plain.

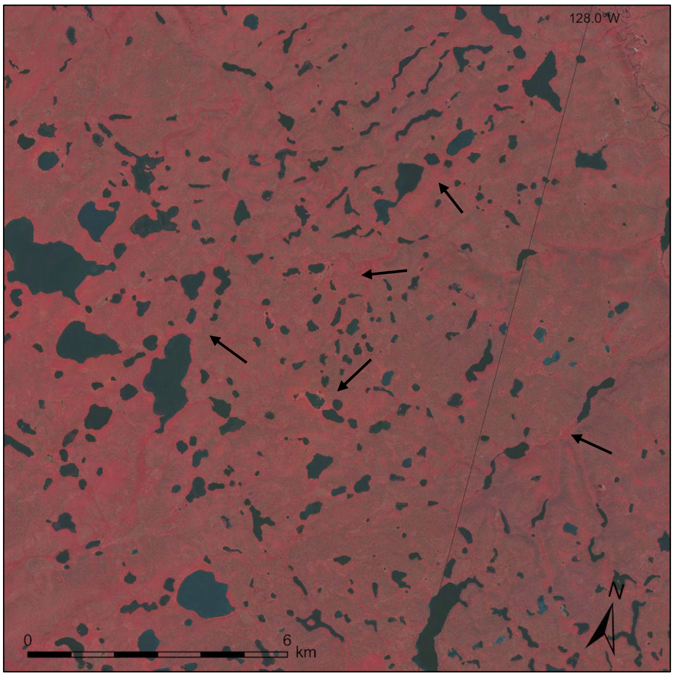

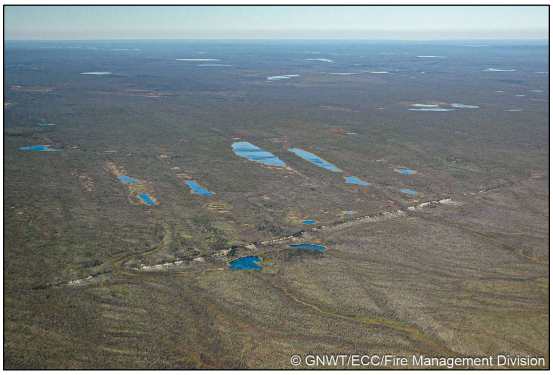

NWT2005-08-14-001DSC_0293.jpg

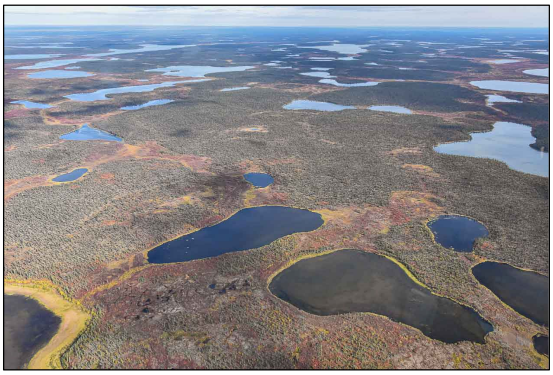

20210827_120812_NIKON_NSL_0427.jpg

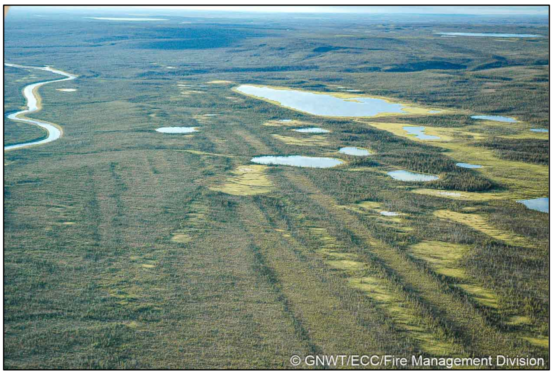

NWT2005-07-24-001DSC_0584.jpg

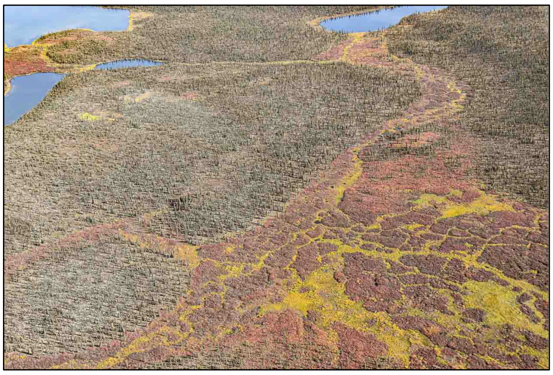

20210827_121652_NIKON_NSL_0436.jpg

East Banks Hills (EBH)

Northern Arctic

14,493 km²

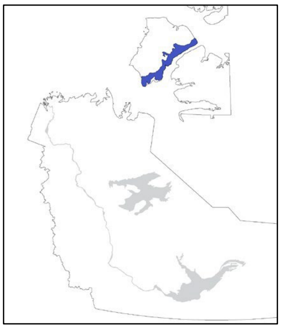

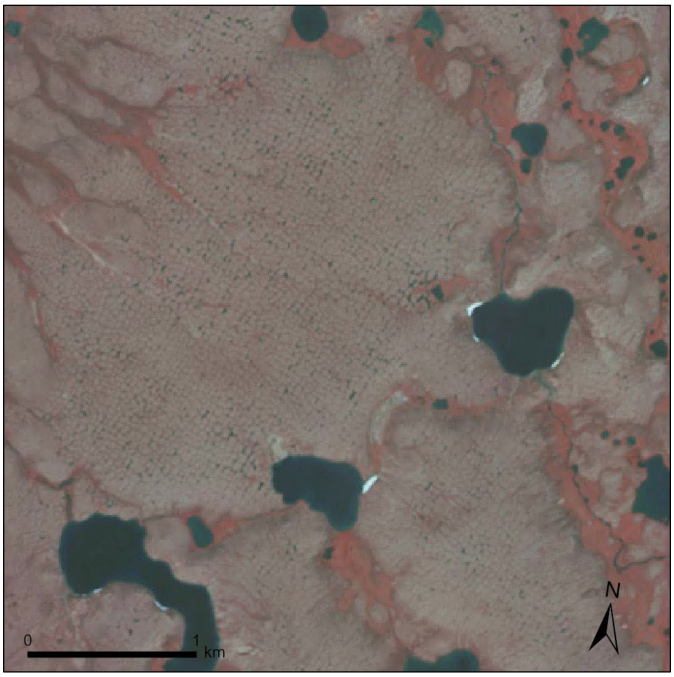

Sentinel-2 imagery of polygonal terrain and polygonal pattern ponding on hill tops and slopes in the East Banks Hills.

NWT_RD2011-07-23DSC_0414.jpg

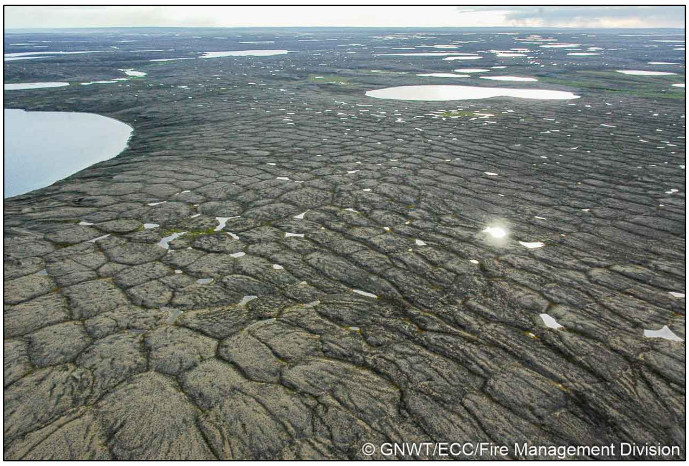

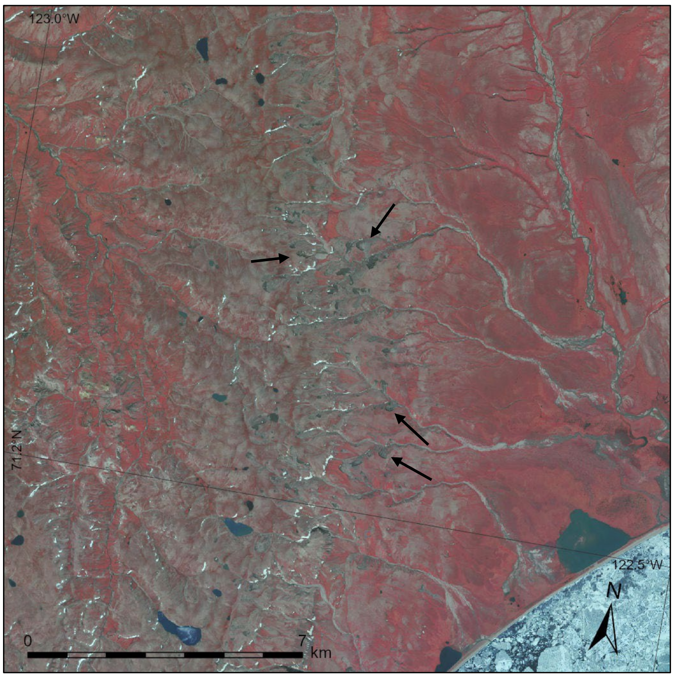

Sentinel-2 imagery of retrogressive thaw slumps in the East Bank Hills.

NWT_RD2011-07-23DSC_0180.jpg

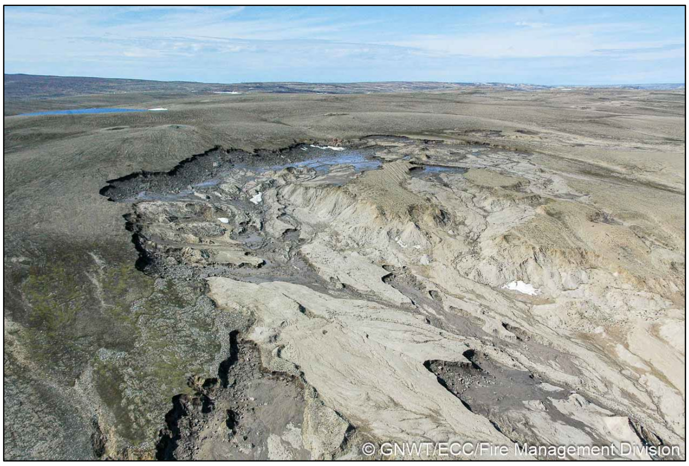

Great Slave Lowland (GSL)

Taiga Shield - High Boreal

11,040 km²

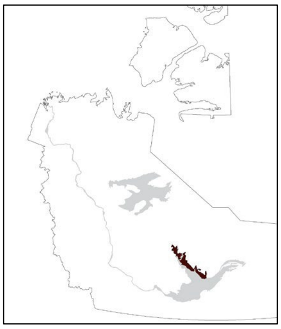

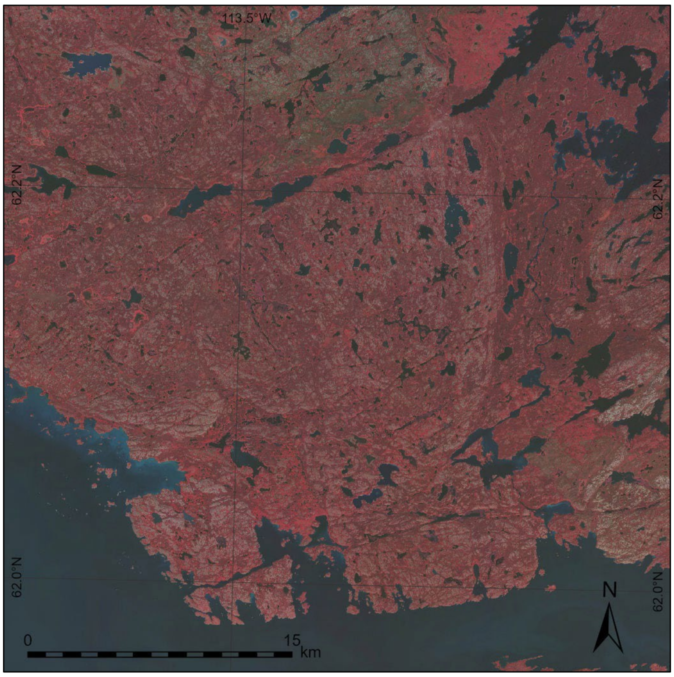

Sentinel-2 imagery of the bedrock dominated and lake-rich Great Slave Lowlands.

20200921_172900_NIKON_NSL_0185.jpg

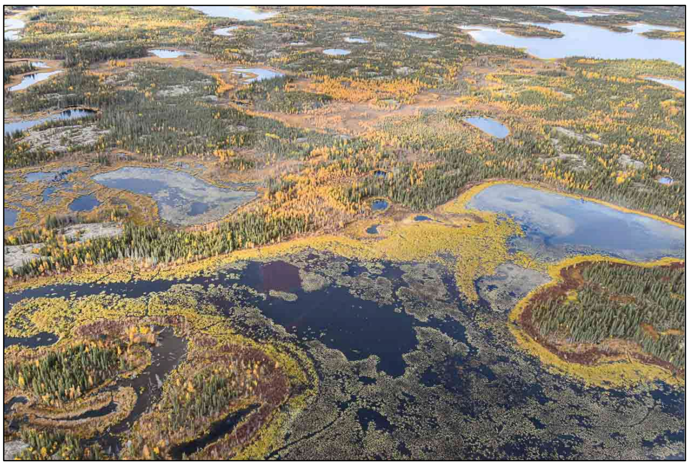

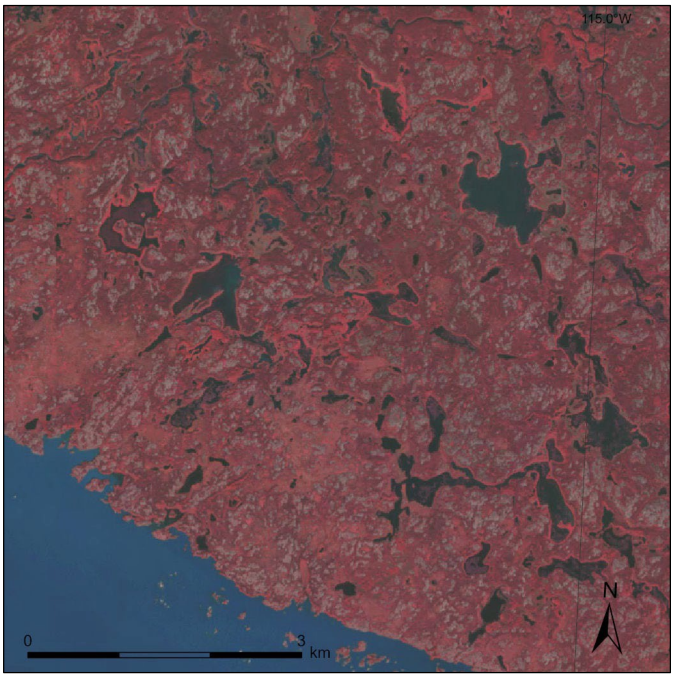

Sentinel-2 imagery of ramparted-lake lithalsa (thaw lake) complexes, and discrete patches of organic terrain in the Great Slave Lowlands.

20200921_172858_NIKON_NWH_4592.jpg

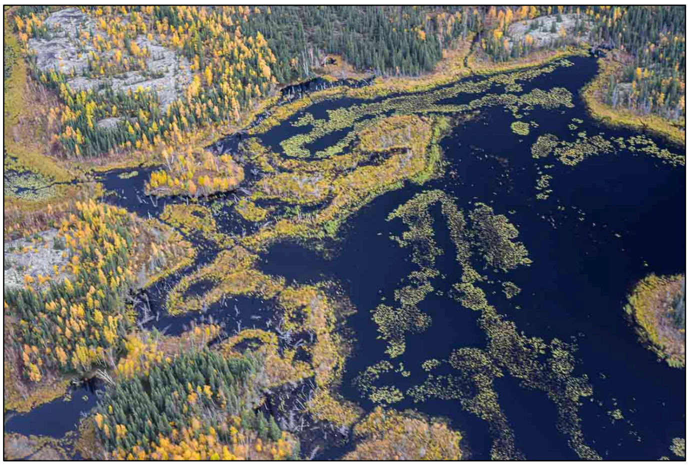

Keller Plain (KP)

Taiga Plains - Low Subarctic

14,494 km²

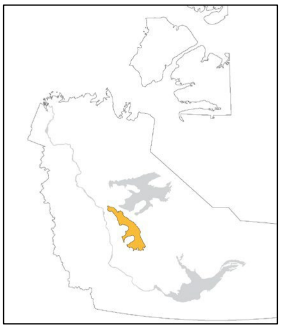

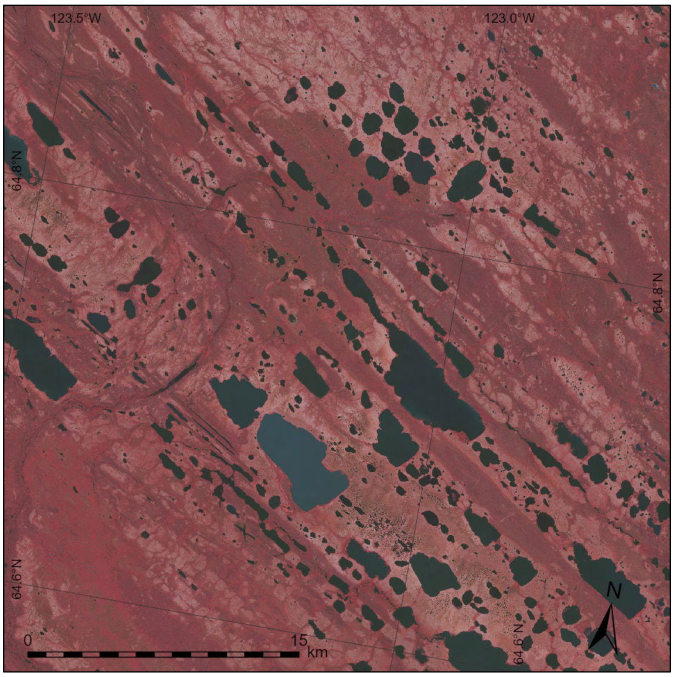

Sentinel-2 imagery of terrain aligned (glacially fluted), geometric lakes and intervening organic terrain with patterned ponding (string bog & undifferentiated) in the Keller Plains.

NWT2005-08-15-001DSC_0340.jpg

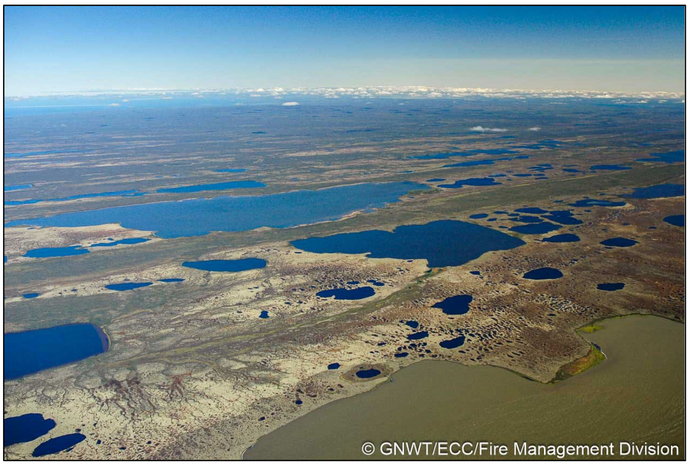

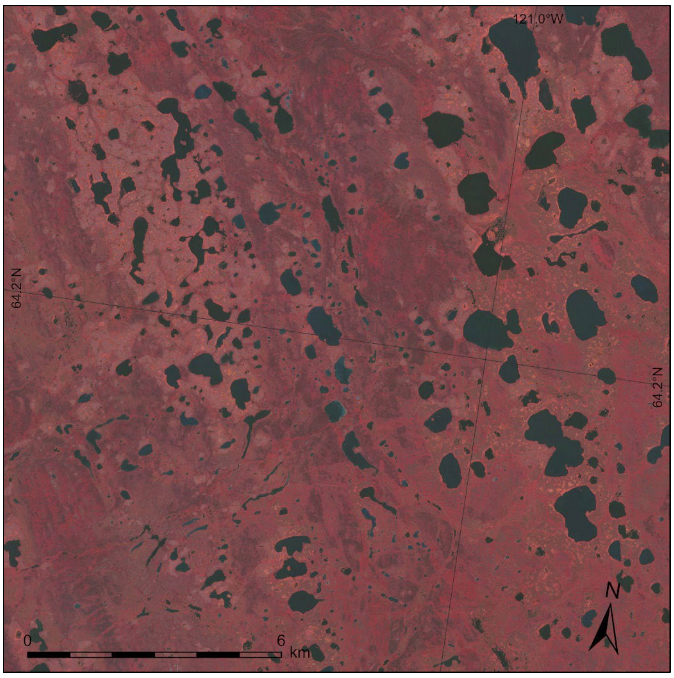

Sentinel-2 imagery of peatlands with collapse scars and patterned ponding (undifferentiated) in the Keller Plains.

NWT2005-08-09-001DSC_0395.jpg

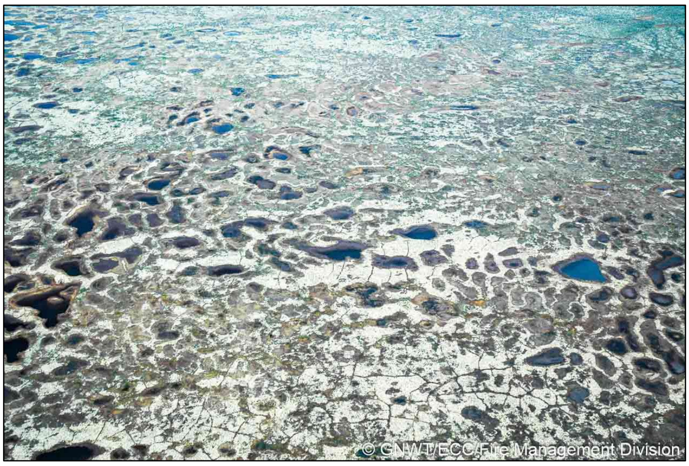

Mackenzie Foothills (MF)

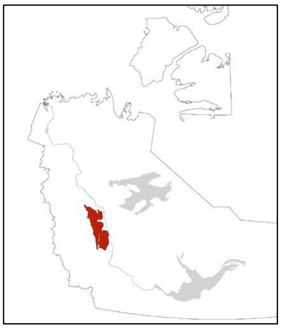

Taiga Cordillera - Low Subarctic

14,000 km²

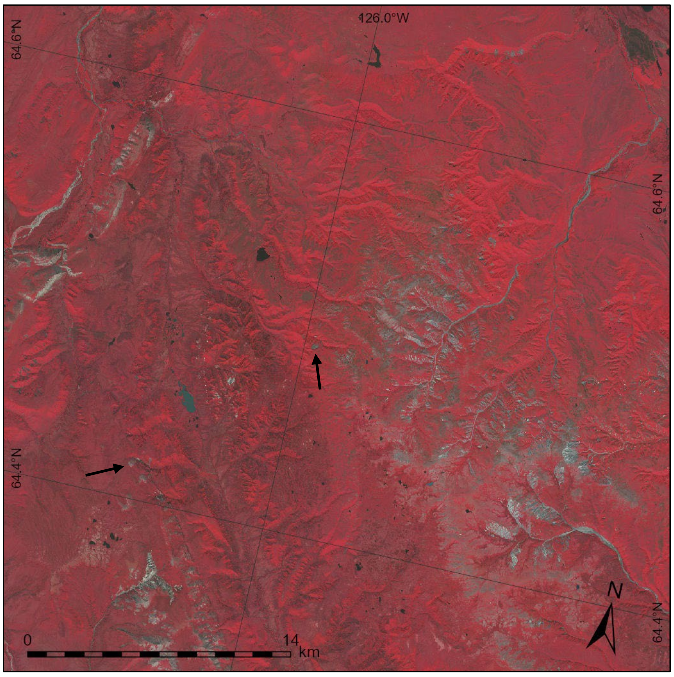

Sentinel-2 imagery of gullied terrain with shallow slides and thaw slumps (black arrows) in Mackenzie Foothills.

20200806_104751_NIKON_NWL_0083.jpg

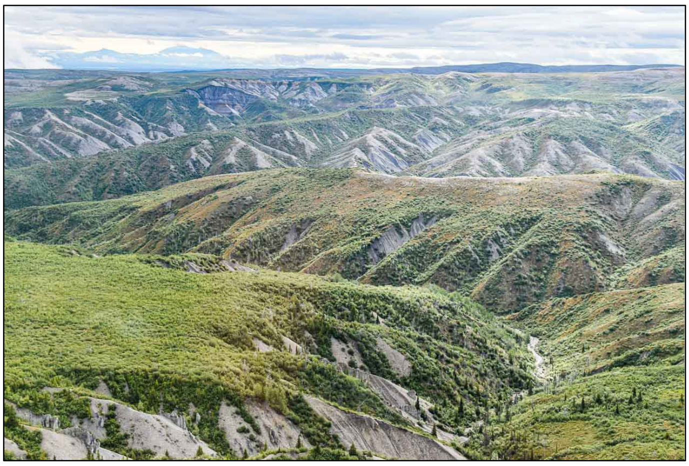

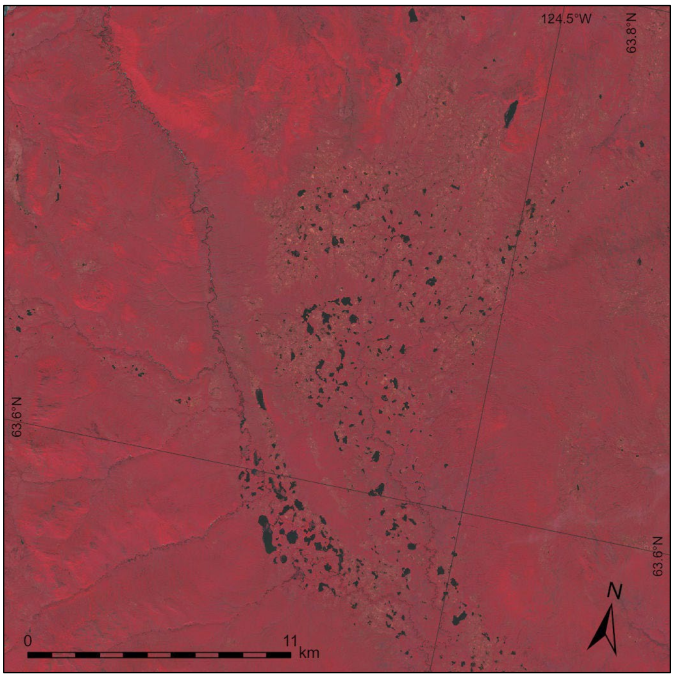

Sentinel-2 imagery of peatlands with collapse scars, thaw ponds (patterned ponds undifferentiated) and ramparted lakes in Mackenzie Foothills.

20200808_130059_NIKON_NWL_0012.jpg

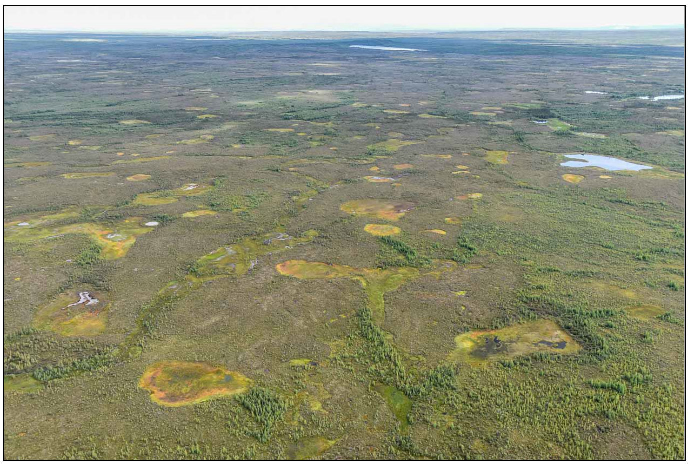

North Mackenzie Plain (MNP)

Taiga Plains - Low Subarctic

16,016 km²

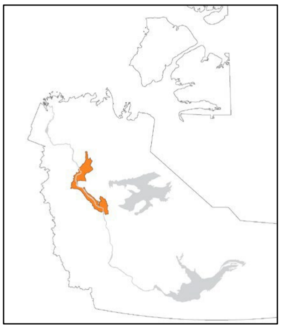

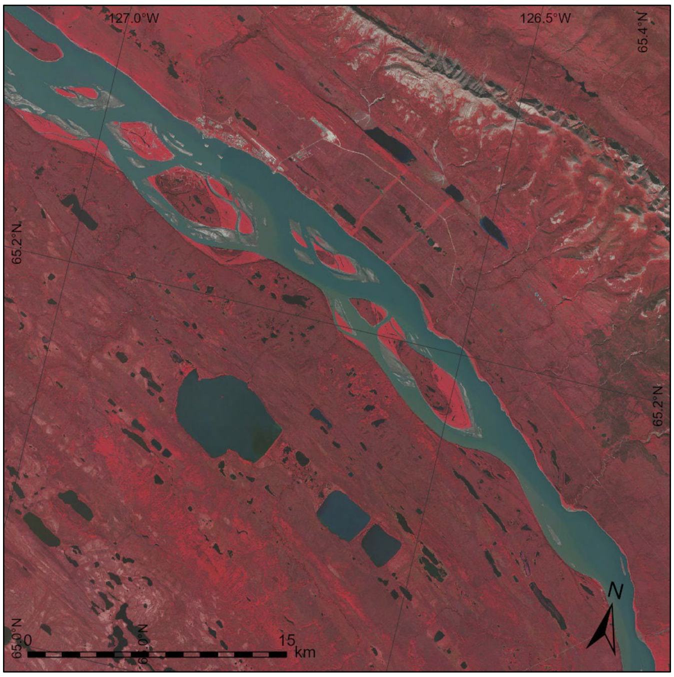

Sentinel-2 imagery of terrain aligned (glacially fluted) and geometric lakes near Norman Wells in the North Mackenzie Plains.

NWT2005-08-15-001DSC_0047.jpg

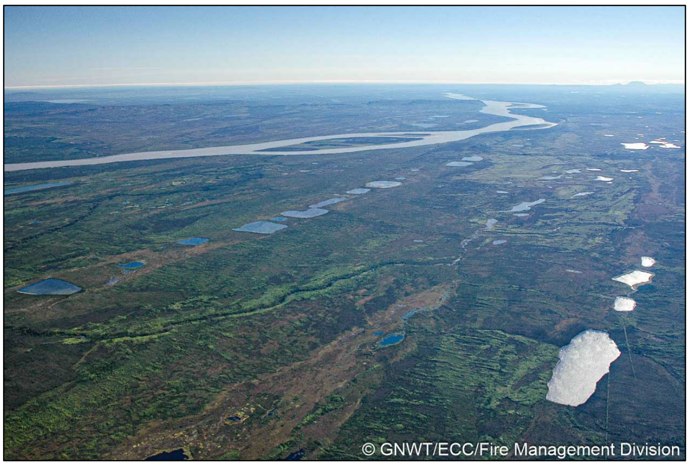

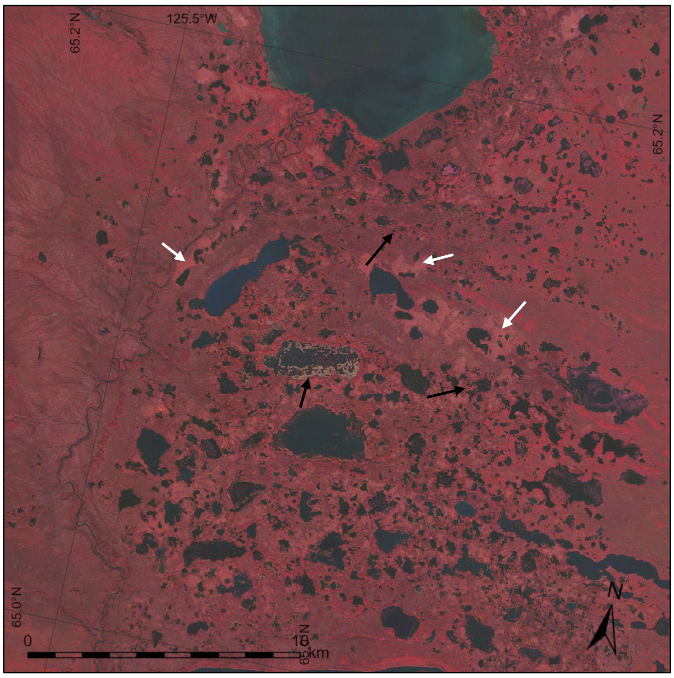

Sentinel-2 imagery of ramparted lake-lithalsa complexes (black arrows), numerous thaw ponds (patterned ponds undifferentiated), and peatlands (white arrows) near Brackett Lake in the North Mackenzie Plains.

20210718_100535_NIKON_NSL_0604.jpg

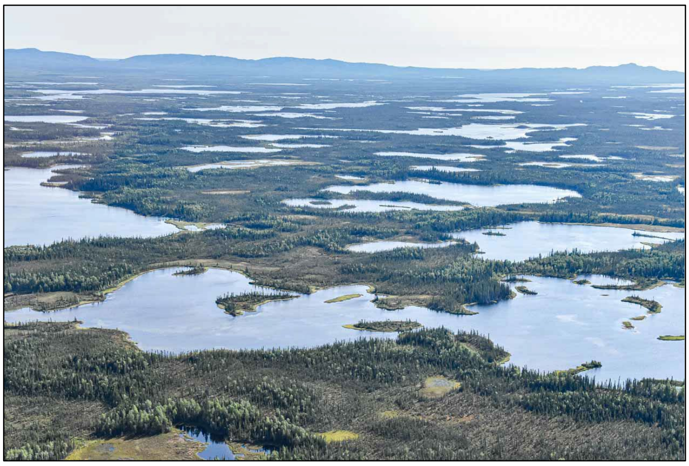

Richardson Plateau (RP)

Tundra Cordillera - High Subarctic

4,195 km²

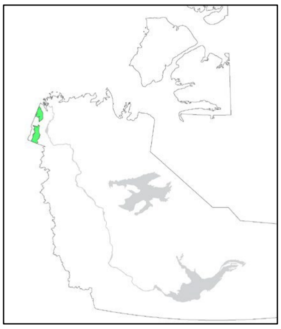

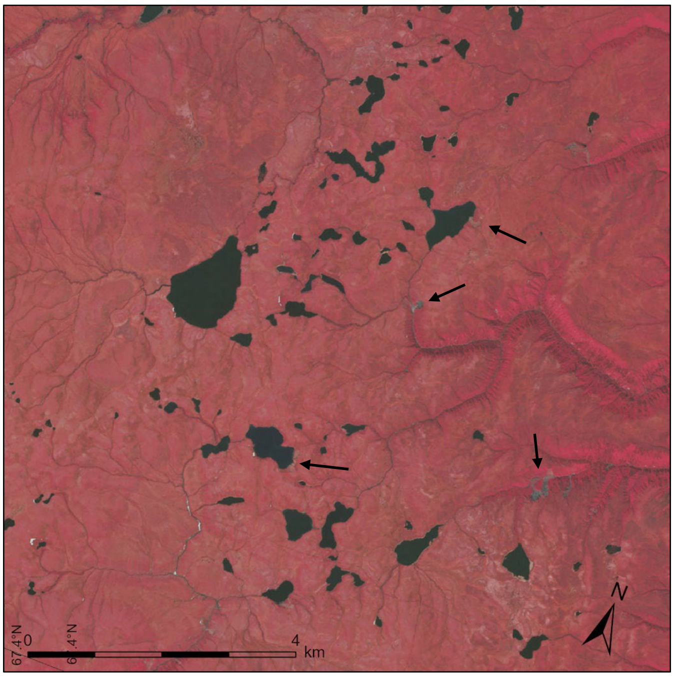

Sentinel-2 imagery of thaw-slump affected lakes and fluvial networks (black arrows), and gullied valleys in the Richardson Plateau.

20210910_114149_NIKON_BDL_0390.jpg

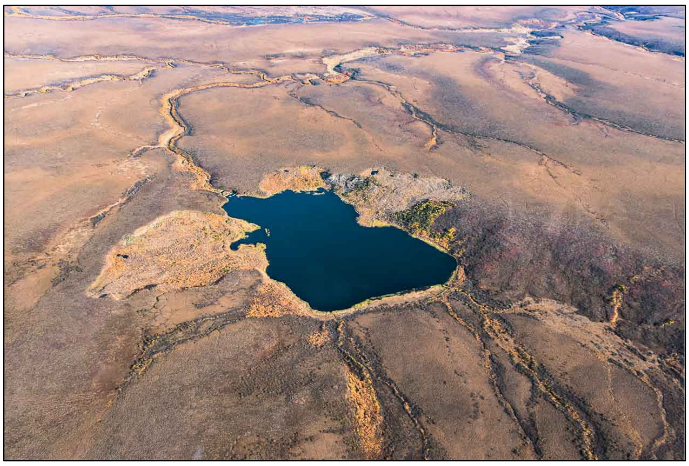

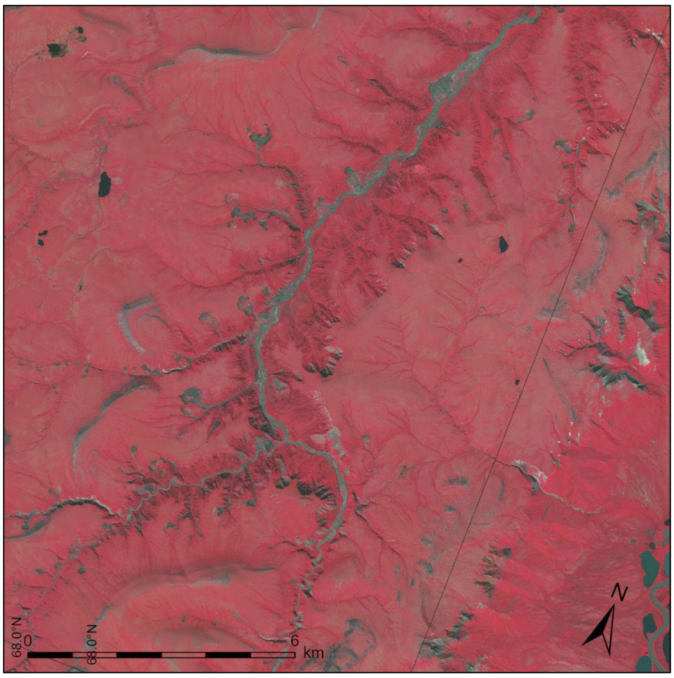

Sentinel-2 imagery of retrogressive thaw slumps, including mega slumps and gullied terrain along the incised fluvial network of the Willow River and tributaries in the Richardson Plateau.

20200823_111337_SONY_DSC04915.jpg

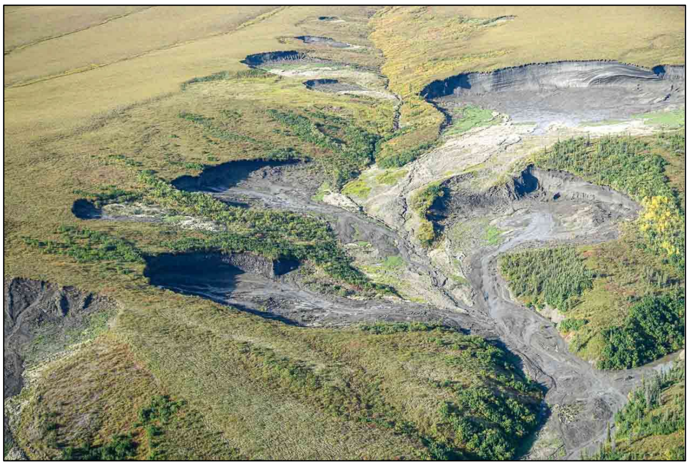

Travaillant Upland (TU)

Taiga Plains - High Subarctic

26,389 km²

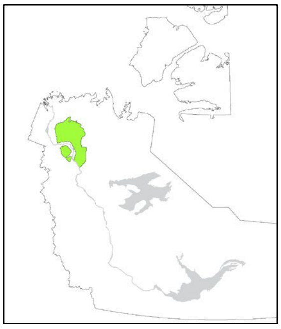

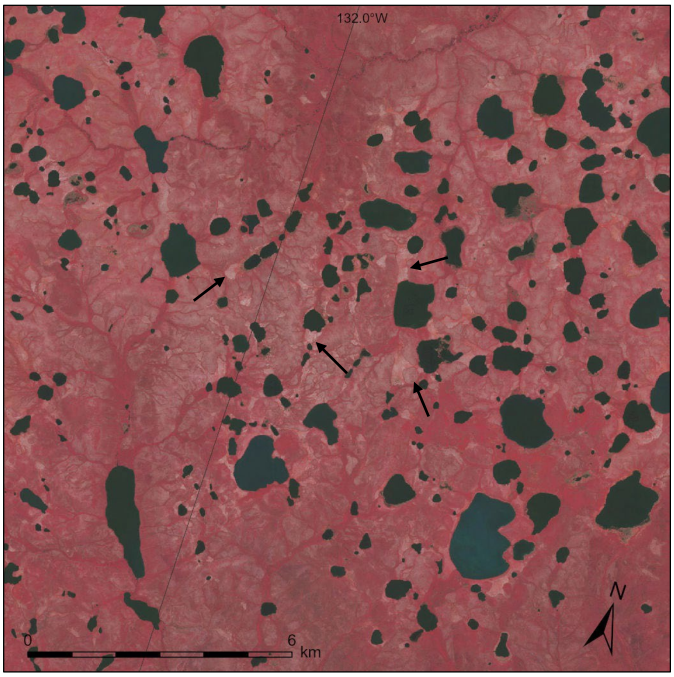

Sentinel-2 imagery showing polygonal peat plateaus (black arrows) in Travaillant Upland.

NWT2005-08-14-001DSC_0165.jpg

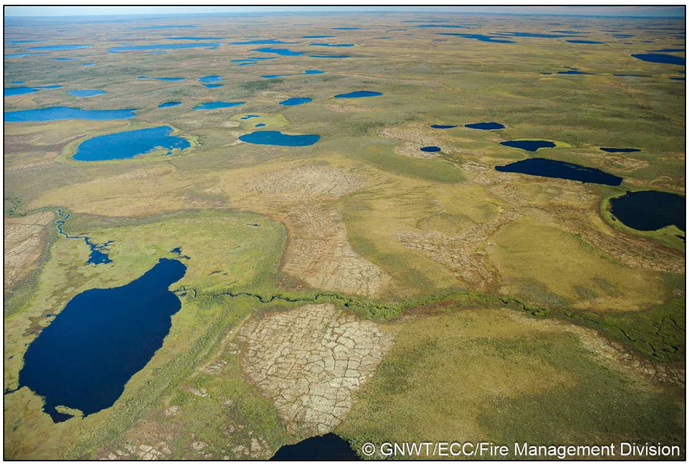

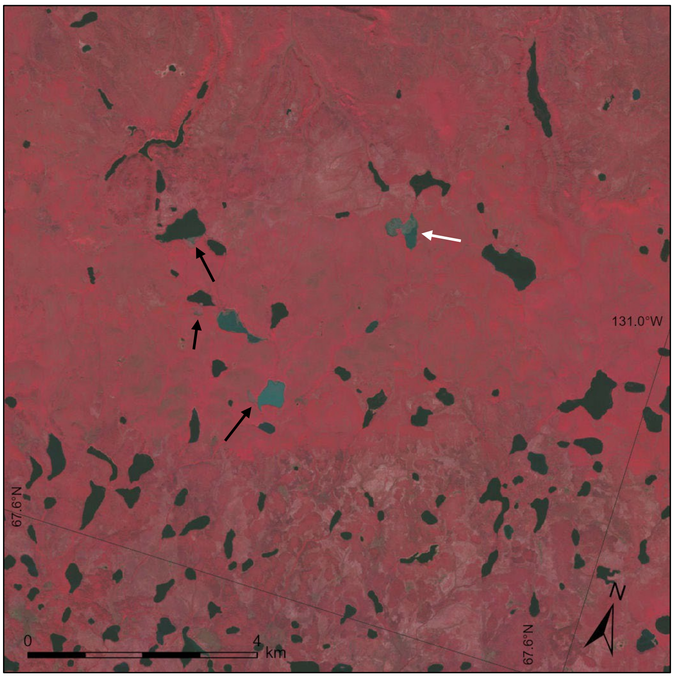

Sentinel-2 imagery of retrogressive thaw slumps (black arrows) along the shorelines of lakes in Travaillant Upland, including a photograph of a mega slump that has infilled a small lake (white arrow).

20210914_150105_NIKON_BDL_0387.jpg

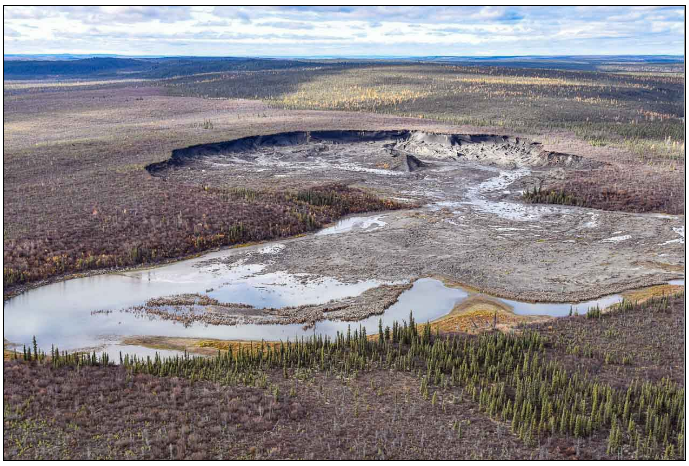

Tuktoyaktuk Coastal Plain (TCP)

Tundra Plains - Southern Arctic

14,816 km²

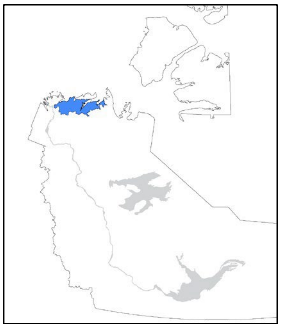

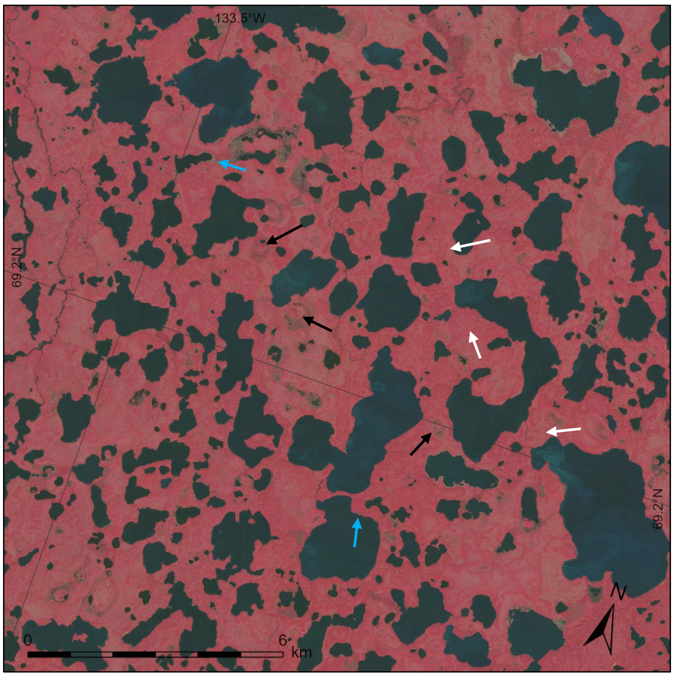

Sentinel-2 imagery of pingos (black arrows and photo bottom row, left), involuted terrain (white arrows and photo bottom row, right) and retrogressive thaw slumps (blue arrows and photo below) in the Tuktoyaktuk Coastal Plains.

20200821_133515_NIKON_INU_0920.jpg

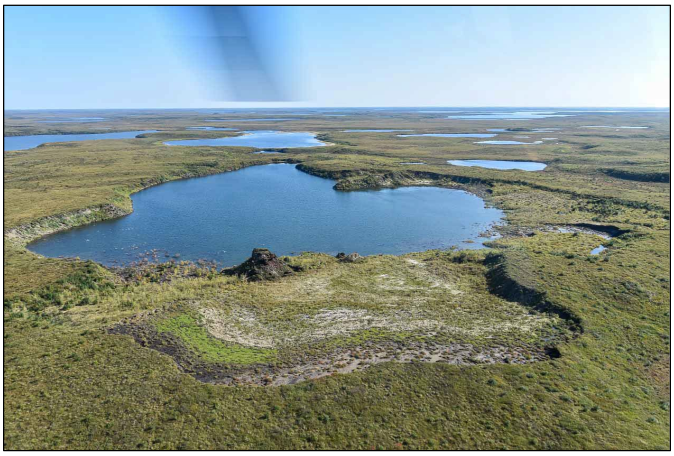

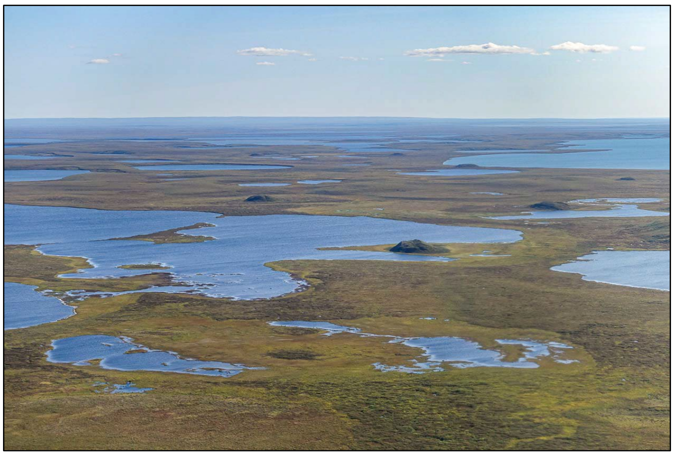

20200822_165148_SONY_DSC04143.jpg

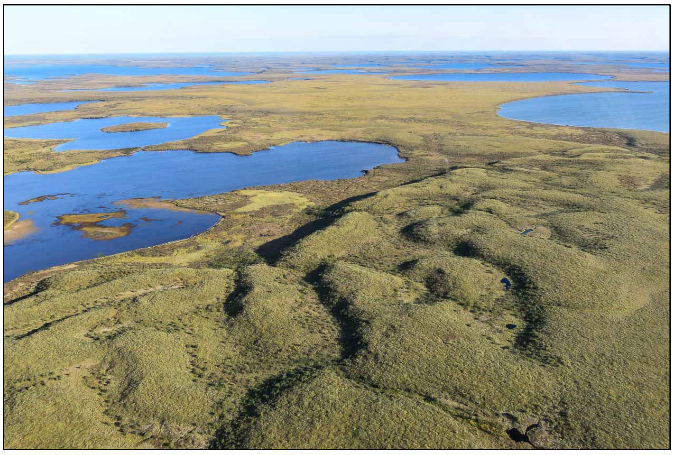

20200821_194022_NIKON_INU_0339.jpg

Tuktoyaktuk Peninsula Coastal Lowland (TPCL)

Tundra Plains – Southern Arctic

2,550 km²

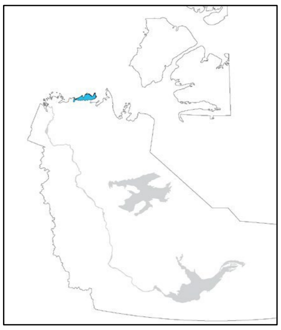

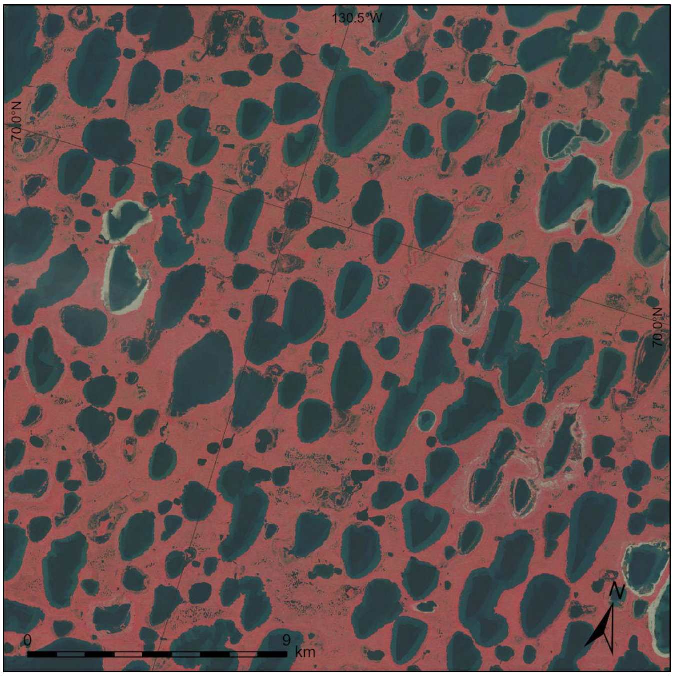

Sentinel-2 imagery of terrain aligned (oriented), geometric lakes with littoral terraces in the Tuktoyaktuk Peninsula Coastal Lowland.

20210909_180043_NIKON_BDL_0893.jpg

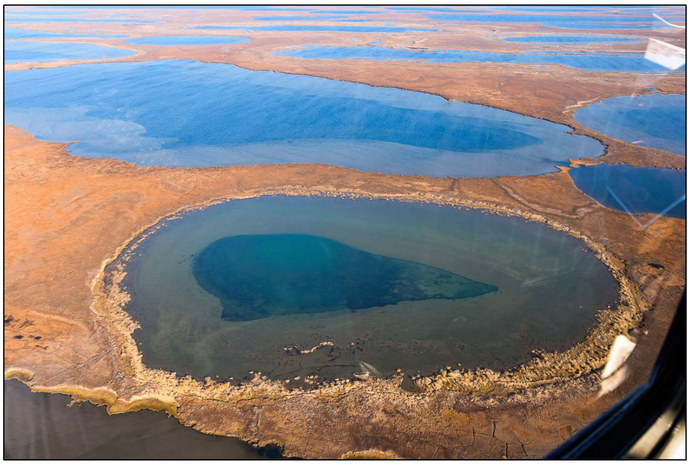

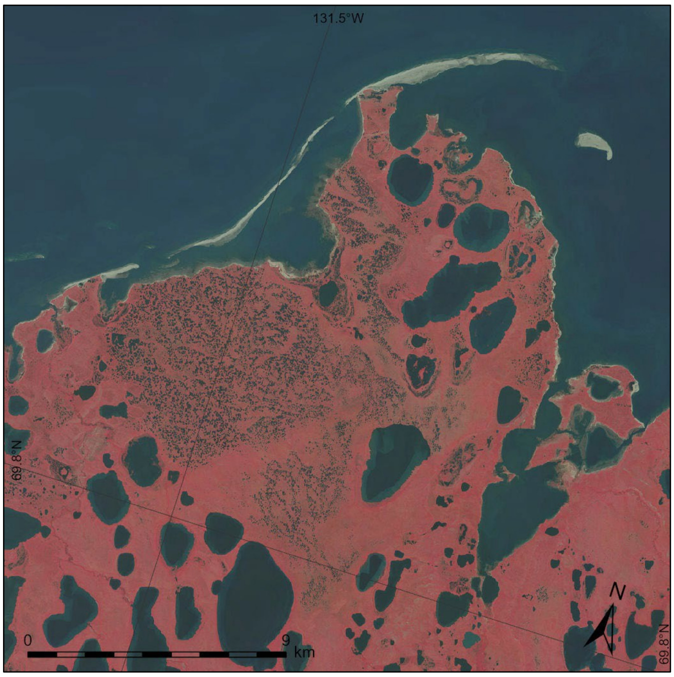

Sentinel-2 imagery of extensive polygonal patterned ponding in the Tuktoyaktuk Peninsula Coastal Lowland.

20210909_175423_SONY_DSC04457.jpg

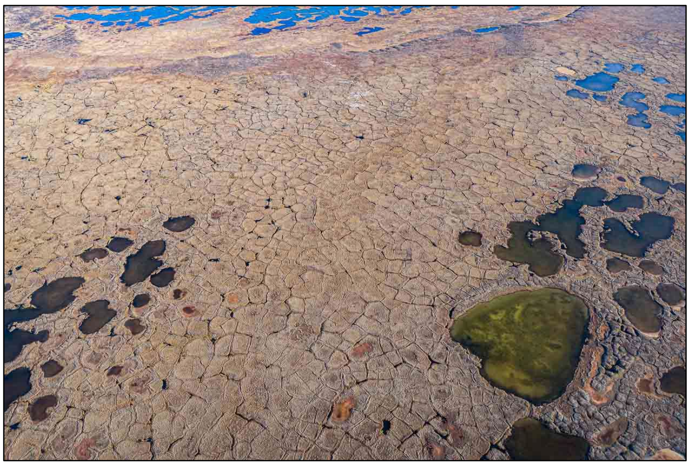

References

1. Kokelj, S. V. *et al.* The Northwest Territories Thermokarst Mapping Collective: A northern-driven mapping collaborative toward understanding the effects of permafrost thaw. *Arctic Science* AS-2023-0009 (2023) doi:10.1139/AS-2023-0009.
2. Ecosystem Classification Group. *Ecological Regions of the Northwest Territories – Taiga Plains*. (Department of Environment and Natural Resources, Government of the Northwest Territories, Yellowknife, NT, Canada, 2007).
3. Ecosystem Classification Group. *Ecological Regions of the Northwest Territories – Taiga Shield*. (Department of Environment and Natural Resources, Government of the Northwest Territories, Yellowknife, NT, Canada, 2008).
4. Ecosystem Classification Group. *Ecological Regions of the Northwest Territories – Cordillera*. (Department of Environment and Natural Resources, Government of the Northwest Territories, Yellowknife, NT, Canada, 2010).
5. Ecosystem Classification Group. *Ecological Regions of the Northwest Territories – Southern Arctic*. (Department of Environment and Natural Resources, Government of the Northwest Territories, Yellowknife, NT, Canada, 2012).
6. Ecosystem Classification Group. *Ecological Regions of the Northwest Territories – Northern Arctic*. (Department of Environment and Natural Resources, Government of the Northwest Territories, Yellowknife, NT, Canada, 2013).
7. Lewkowicz, A. G. *Glossary of Permafrost Science and Engineering*. (University of Toronto Press, 2025). doi:10.3138/cpa-gpse.
8. Muñoz Sabater, J. ERA5-Land monthly averaged data from 1950 to present. Copernicus Climate Change Service (C3S) Climate Data Store (CDS) https://doi.org/10.24381/cds.68d2bb30 (2019).
9. Government of Northwest Territories (GNWT). *Fire History*. https://www.geomatics.gov.nt.ca/en/fire-history.
10. Amatulli, G., McInerney, D., Sethi, T., Strobl, P. & Domisch, S. Geomorpho90m - Global High-Resolution Geomorphometry Layers. *Distributed by OpenTopography* https://doi.org/10.5069/G91R6NPX (2020) doi:10.5069/G91R6NPX.
11. Donchyts, G. *et al.* Global 30m height above the nearest drainage (HAND). *Geophysical Research Abstracts* **Geophysical Research Abstracts, Vol. 18, EGU2016-17445-3, 2016, EGU General Assembly**, (2016).
12. Amatulli, G. *et al.* Hydrography90m: a new high-resolution global hydrographic dataset. *Earth Syst. Sci. Data* **14**, 4525–4550 (2022).
13. *Topographic Data of Canada - CanVec Series. Lakes, Rivers and Glaciers in Canada - Hydrographic Features - 1:250k*. https://open.canada.ca/data/en/dataset/9d96e8c9-22fe-4ad2-b5e8-94a6991b744b.
14. *Canadian Digital Elevation Model, 1945-2011 (CDEM)*. https://open.canada.ca/data/en/dataset/7f245e4d-76c2-4caa-951a-45d1d2051333.
15. O'Neill, H. B., Wolfe, S. A. & Duchesne, C. New ground ice maps for Canada using a paleogeographic modelling approach. *The Cryosphere* **13**, 753–773 (2019).
16. Obu, J., Westermann, S., Kääb, A. & Bartsch, A. Ground Temperature Map, 2000-2016, Northern Hemisphere Permafrost. 40 data points PANGAEA https://doi.org/10.1594/PANGAEA.888600 (2018).
